# Comparison of Different Labeling Techniques for the LC-MS Profiling of Human Milk Oligosaccharides

**DOI:** 10.3389/fchem.2021.691299

**Published:** 2021-09-13

**Authors:** Yinzhi Lang, Yongzhen Zhang, Chen Wang, Limei Huang, Xiaoxiao Liu, Ni Song, Guoyun Li, Guangli Yu

**Affiliations:** ^1^ Key Laboratory of Marine Drugs, Ministry of Education, Shandong Provincial Key Laboratory of Glycoscience and Glycotechnology, School of Medicine and Pharmacy, Ocean University of China, Qingdao, China; ^2^ Laboratory for Marine Drugs and Bioproducts, Qingdao National Laboratory for Marine Science and Technology, Qingdao, China

**Keywords:** human milk oligosaccharides, labeling, 2-aminobenzoic acid, HILIC-MS, PGC-MS

## Abstract

Human milk oligosaccharides (HMOs) exhibit various biological activities for infants, such as serving as prebiotics, blocking pathogens, and aiding in brain development. HMOs are a complex mixture of hetero-oligosaccharides that are generally highly branched, containing multiple structural isomers and no intrinsic chromophores, presenting a challenge to both their resolution and quantitative detection. While liquid chromatography-mass spectrometry (LC-MS) has become the primary strategy for analysis of various compounds, the very polar and chromophore-free properties of native glycans hinder their separation in LC and ionization in MS. Various labeling approaches have been developed to achieve separation of glycans with higher resolution and greater sensitivity of detection. Here, we compared five commonly used labeling techniques [by 2-aminobenzamide, 2-aminopyridine, 2-aminobenzoic acid (2-AA), 2,6-diaminopyridine, and 1-phenyl-3-methyl-5-pyrazolone] for analyzing HMOs specifically under hydrophilic-interaction chromatography-mass spectrometry (HILIC-MS) conditions. The 2-AA labeling showed the most consistent deprotonated molecular ions, the enhanced sensitivity with the least structural selectivity, and the sequencing-informative tandem MS fragmentation spectra for the widest range of HMOs; therefore, this labeling technique was selected for further optimization under the porous graphitized carbon chromatography-mass spectrometry (PGC-MS) conditions. The combination strategy of 2-AA labeling and PGC-MS techniques provided online decontamination (removal of excess 2-AA, salts, and lactose) and resolute detection of many HMOs, enabling us to characterize the profiles of complicated HMO mixtures comprehensively in a simple protocol.

## Introduction

Human milk oligosaccharides (HMOs) have been assigned to a variety of important biological functions for infants (Newburg, 2013; Bode, 2015; Wicinski et al., 2020), such as prevention of pathogens binding to epithelial cell surfaces (Hester et al., 2013; Gonia et al., 2015; Triantis et al., 2018; Ha et al., 2020), functioning as prebiotics (Boehm et al., 2005; Marcobal and Sonnenburg, 2012; Underwood et al., 2015; Walsh et al., 2020), and enhancing brain development (Oliveros et al., 2018; Docq et al., 2020; Wu et al., 2020). Characterizing the structure(s) and concentration(s) of the isolated specific HMO(s) is essential to elucidate its (or their) molecular mechanisms involved in *in vitro* studies. For understanding the difference of HMOs in different populations and the changes of HMOs at different lactations, component analysis of a whole HMO mixture is necessary. However, HMOs are hetero-oligosaccharides composed of different glycosidic-linked glucose, galactose, *N*-acetyl-glucosamine, fucose, and *N*-acetyl-neuraminic acid residues (Kunz et al., 2000; Bode, 2006), where the five monosaccharides can be linked in various ways through at least 12 α- and β-glycosidic linkages, resulting in a structurally complex array of linear, branched, and isomeric structures (Bode, 2012; Smilowitz et al., 2014). The structural diversity and complexity present a challenge to their resolute separation and detection.

Over the years, many analytical techniques have been applied to analyze HMOs as reviewed previously (Mantovani et al., 2016). Nuclear magnetic resonance spectroscopy (NMR) and off-line mass spectrometry (MS) have previously been the major methods for HMO structural elucidations (Chai et al., 2005). High-pH anion-exchange chromatography (HPAEC) coupled with pulsed amperometric detection (PAD) required the preliminary sample preparation to remove any other biomolecules (Thurl et al., 1997; Coppa et al., 1999). Liquid chromatography (LC) and capillary electrophoresis (CE) coupled with UV adsorption or laser-induced fluorescence (LIF) detection have previously been the major methods for HMO (relatively) quantification analysis by tagging the native oligosaccharides with chromophores and fluorophores (Ruhaak et al., 2010). Even though these methods permit higher UV sensitivity or fluorescence detection, they do not provide specific structural information, and this is a key point due to the highly complex nature of milk glycan mixtures. Furthermore, these methods are severely limited by the small number of HMO standards commercially available. As a consequence, the combination of analytical separation (e.g., LC) and MS has been extensively and successfully used for oligosaccharide compositional profiling analysis in various milk samples (Tao et al., 2008; Tao et al., 2009; Wu et al., 2010; Wu et al., 2011). Since the native glycan analysis was ambiguous due to the separation of anomers under LC conditions, Carlito B. Lebrilla and his colleagues employed a mild reduction reaction to transform the native oligosaccharides to their alditol forms to simplify the chromatograms and determined many oligosaccharides in bovine milk and human milk samples (Tao et al., 2008; Tao et al., 2009; Wu et al., 2010; Wu et al., 2011). This method allows identification and relatively quantification based on the MS signal; however, the very polar hydrophobic groups or the lack of hydrophobic groups (e.g., chromophores, fluorophores, etc.) is always associated with a weak volatility during electrospray ionization (ESI), especially for some large oligosaccharides, which may thus lead to insufficient ionization of such oligosaccharide alditols in MS. Therefore, future research is needed to explore suitable labeling approaches for HMOs to specifically enhance the analytical performance of LC-MS techniques (Ruhaak et al., 2010).

As mentioned above, a few labeling approaches have previously been developed for optical detection of HMOs when coupled with CE or LC analytical separations, for example, labeling the HMOs with 2-aminoacridone (2-AMAC), 1-phenyl-3-methyl-5-pyrazolone (PMP), 2-aminopyridine (2-AP), 2-aminobenzoic acid (2-AA), 2-aminobenzamide (2-AB), 8-aminopyrene-1,3,6-trisulfonate (APTS), etc. (Song et al., 2002; Domann et al., 2007; Asakuma et al., 2008; Leo et al., 2010; Mariño et al., 2011). The most common labeling reaction employed is reductive amination, which can attach a fluorescence label containing amino groups to the anomeric center of a native glycan’s reducing terminus. It is usually performed in a dimethyl sulfoxide solution containing acetic acid (or citric acid), for example, 2-AB, 2-AA, 2,6-diaminopyridine (DAP), AEAB, 2-AMAC, 2-AP, APTS, etc. (Anumula, 1994; Bigge et al., 1995; Morelle et al., 2005; Xia et al., 2005; Anumula, 2006; Song et al., 2009; Galeotti et al., 2012; Kishimoto et al., 2020; Kinoshita et al., 2021). 2-AB and 2-AP have been widely applied in hydrophilic-interaction chromatography and mass spectrometry (HILIC-MS) profiling, and databases for structural assignments based on standardized elution positions have been developed (Morelle et al., 2005; Takegawa et al., 2005; Royle et al., 2008; Kozak et al., 2015). DAP, with two aromatic amine groups, has been developed as a versatile tag for the combination of structural characterization and biomolecular interaction analysis in glycan array fabrication studies (Xia et al., 2005). Anumula developed an alternative mild reaction for 2-AA specifically via acetate-borate-buffered methanol solution (Anumula, 2006). 2-AA bears one negative charge specifically, showing versatility in CE separations as well as matrix-assisted laser desorption/ionization (MALDI) analysis. 2-AA derivatization was reported to allow for simultaneous MALDI analysis of neutral and acidic N-glycans (Anumula and Dhume, 1998). However, whether such benefit remains for its application to ESI-MS analysis of glycans has rarely been discussed. Another widely employed derivatization reaction, Michael addition for conjugation of PMP (without the primary amine group), was performed under alkaline conditions (Strydom, 1994; You et al., 2008; Wang et al., 2018). PMP labeling shows high sensitivity by UV absorption and has been expected to reduce the risk of losing sialic acids (Saba et al., 1999; Wang et al., 2013). These labeling approaches have also been widely applied to *N*-glycan analysis by improving the optical detection and furthermore have been reported to be capable of increasing the sensitivity of MS detection (Anumula, 1994; Bigge et al., 1995; Morelle et al., 2005; Xia et al., 2005; Anumula, 2006; Song et al., 2009; Galeotti et al., 2012; Kishimoto et al., 2020; Kinoshita et al., 2021).

To allow compatibility with the LC-MS system, the reaction mixtures usually need to be purified from excess salts and fluorescent tags by solid-phase extraction (SPE) (Redmond and Packer, 1999; Zhang et al., 2014) or liquid-liquid extraction (Yuen et al., 2002). While this process increases glycan purity and facilitates in-depth qualification analysis by following the LC-MS technique, it may introduce a selective loss of glycans for samples that are a mix of structurally diverse glycans (Blank et al., 2011). For the LC separation, HILIC has been widely utilized to separate the reductive amination-derivatized glycans (Ahn et al., 2010; Lauber et al., 2015; Cesla et al., 2016; Kinoshita et al., 2021), and reversed-phase chromatography (RPC) has been employed to separate the PMP-derivatized glycans (Melmer et al., 2011; Zauner et al., 2012). Surprisingly, porous graphitic carbon chromatography (PGC) has rarely been combined with chromophore-derivatization methods for glycan analysis, although it has been widely applied to LC-MS analysis of HMO reduced alditols (Tao et al., 2008; Tao et al., 2009; Wu et al., 2010; Wu et al., 2011). For the MS detection, David J. Harvey systematically studied the effect of different derivatives of six purchased *N*-glycans on electrospray ionization (ESI) sensitivity and collision-induced dissociation (CID) fragmentation using an LC-offline Q-TOF mass spectrometer (Harvey, 2000). The in-line LC-MS mode provided powerful resolution and quantitative detection for compounds; however, the components of injected analytes and mobile phases need to be considered as vital factors that can carry the interfered sodium adduct ions which would result in the varying detection sensitivity and identification ambiguity.

Several reviews systematically evaluated and compared the influence of different *N*-glycan derivatives on the MS performance at both off-line or in-line modes (Suzuki et al., 1996; Harvey, 2000; Saba et al., 2001; Gao et al., 2003; Lattova et al., 2005; Pabst et al., 2009; De Leoz et al., 2020), and many conclusive results were obtained, providing useful guidance on selection of suitable analytical strategies for *N*-glycan analysis. For analysis of HMOs, a number of analytical techniques have been developed, and several reviews provided comparative evaluation on the quantification analysis of HMOs (Ninonuevo et al., 2006; Bao and Newburg, 2008; Mantovani et al., 2016; Grabarics et al., 2017; Tonon et al., 2019; van Leeuwen, 2019; Auer et al., 2021). Regarding the compositional profiling analysis of HMO subjects, however, comparative studies that focused on LC-MS-based HMO labeling strategies are limited (Mariño et al., 2011). This study aimed to compare how five different widely used labeling techniques (2-AA, 2-AB, 2-AP, DAP, and PMP), two sample pretreatment procedures (SPE and none), and two LC-MS techniques (HILIC-MS and PGC-MS) can influence the MS performance and the compositional profiling analysis results of a HMO mixture. This fundamental study will support future research to discover HMO biomarkers relevant to infant-protective functions and to elucidate the maternal expression dynamics of HMOs associated with the lactation cycle.

## Materials and Methods

### Chemicals and Reagents

Mature milk samples from 13 healthy lactating women volunteers were pooled as one sample (Qingdao, Shandong, China). This study was carried out in accordance with the recommendations of the Scientific Ethics Special Committee guidelines of Ocean University of China. The labels (2-AB, 2-AP, 2-AA, DAP, and PMP) and reducing agents (sodium cyanoborohydride, NaCNBH_3_, and sodium borohydride, NaBH_4_) were purchased from Sigma-Aldrich (Vienna, Austria). Acetonitrile, ammonium formate, ammonium acetate, ammonium bicarbonate, sodium acetate, formic acid, acetic acid, trifluoroacetic acid, dimethyl sulfoxide, and ammonia were obtained from Merck (Darmstadt, Germany). Water was prepared using a Milli-Q® system (Millipore, MA, United States). The self-packed SPE tubes: the bottom frit was loaded to the bottom of the cartridge using a push rod, filling the sorbent, and the top frit was loaded to the top of the cartridge using a push rod. Sorbents: graphitized carbon cartridges (GCCs, 300 mg of bed weight, a 7 ml cartridge volume) were purchased from Grace Davison (IL, United States), and polyamide S6 cartridges (Discovery® DPA-6S, 300 mg, a 7 ml cartridge volume) were purchased from Sigma-Aldrich (MO, United States). Prior to separation, cartridges were activated according to the manufacturer’s protocol.

### Extraction of HMOs

A crude mixture of HMOs was prepared from the pooled human milk sample as described previously (Ward, 2009). Briefly, 2 ml of milk was centrifuged at 4,500 *g* at 4°C for 30 min, and the majority of the fatty layer (upper layer) was discarded. 4 ml of ethanol was then added to the defatted milk (bottom layer), vortexed, and kept at 4°C for overnight. The insoluble protein-rich precipitate was removed by centrifugation at 4,500 *g* at 4°C for 10 min. The oligosaccharide-rich fraction (top layer) was dried *in vacuo* and referred to as C-HMOs.

A refined mixture of HMOs was isolated from the residual peptides and the high amount of lactose by SPE using GCC (Ward, 2009; Blank et al., 2011). Briefly, the C-HMOs were reconstituted in water, loaded on the GCC, and then washed with water containing 0.1% trifluoroacetic acid to remove lactose and salts. HMOs were eluted with 40% acetonitrile/water (v/v) (containing 0.1% trifluoroacetic acid). The pooled eluent was lyophilized and referred to as R-HMOs.

### Derivatization of HMOs

*Labeling by reductive amination.* 2-AB, 2-AP, and DAP derivatives were essentially prepared by the method described by J. C. Bigge et al. (1995). Dried C-HMOs or R-HMOs (1 mg) were dissolved in anhydrous 70% dimethyl sulfoxide: 30% acetic acid (100 μl), followed by NaCNBH_3_ and the labeling reagent (2-AB, 2-AP, or DAP) being added to give final concentrations of 63 mg/ml and 50 mg/ml, respectively. The mixture was heated for 2 h at 65°C. Derivatization of HMOs with 2-AA was carried out as described previously (Anumula, 2014). A solution of 4% sodium acetate (w/v) and 2% boric acid (w/v) in methanol was prepared first. The derivatization reagent was freshly prepared by dissolving 63 mg/ml of NaCNBH_3_ and 44 mg/ml of 2-AA in 1.0 ml of the above methanol-sodium acetate-borate solution. The HMOs (1 mg in 20 μl of water) were mixed with 100 μl of the above 2-AA solution for 1 h at 80°C.

*Labeling by Michael addition.* C-HMOs or R-HMOs (1 mg) were dissolved in 0.3 M NaOH in water (50 μl), mixed with a methanolic solution of PMP (0.5 M, 50 μl), and incubated for 1 h at 70°C (Strydom, 1994). After cooling, the mixture was neutralized by adding 50 μl of HCl solution (0.3 M).

*Derivatization by reduction.* C-HMOs or R-HMOs (1 mg) were reduced to their alditol forms using 1.0 M NaBH_4_ in water (100 μl) and incubated for 1.5 h at 65°C (Lattova et al., 2005). This was designed as a reference method without introducing chromophores.

For derivatizations of C-HMOs, reactions were terminated with 1 ml of water, lyophilized, and used directly for LC-MS analysis.

For derivatizations of R-HMOs, the reaction mixtures were purified by one or two SPE processes prior to LC-MS analysis. The reference R-HMO alditols were isolated from excess salts by GCC SPE, as described for removal of lactose above. For 2-AB-, 2-AP-, DAP-, 2-AA-, and PMP-derivatized HMOs, we employed a tandem SPE method: DPA-6S SPE for removing excess hydrophobic labels and GCC SPE for desalting. The labeling reaction mixture was diluted with 1 ml of 95% acetonitrile/water (v/v), loaded on the DPA-6S cartridge, and washed with the same solvent, and then, labeled HMOs (probably with residual salts) were eluted with 20% acetonitrile/water (v/v). This eluent was then loaded to GCC SPE to remove salts. Briefly, the eluent was dried and reconstituted in 1 ml of water, loaded on the GCC, and then washed with water containing 0.1% trifluoroacetic acid to remove salts. HMOs were eluted with 40% acetonitrile/water (v/v) (containing 0.1% trifluoroacetic acid). Samples were lyophilized before LC-MS analysis.

### LC-MS Analysis of HMO Derivatives

All experiments were performed using a 1,260 series capillary LC system (Agilent Technologies, Inc.) coupled to an LTQ-Orbitrap XL mass spectrometer (Thermo Fisher Scientific). Conditions for ionic strength, pH, and the buffer system were optimized to achieve resolute elution behavior for LC and sensitive ionization efficiency for MS.

The applied ES voltage for both negative and positive modes was 3.0 kV, with a capillary temperature of 275°C. The capillary voltage was set at −41 V with the tube lens voltage of −120 V in the negative mode and +18 V with the tube lens voltage of +95 V in the positive-ion mode. The instrument was operated in the Fourier transform mode with *m/z* ranging from 200 to 3,000 Da. The full MS scan (at a resolution of 60,000) was followed by a data-dependent MS/MS scan of the five most abundant ions in the ion trap. For MS/MS in the ion trap, the normalized collision energy was set to 25 arbitrary units with an automated gain control target value of 1×10^4^.

An Inertsil^®^ Amide column (150 × 0.5 mm, 3 μm, GL Sciences) was used for the HILIC separation. The buffer system for HILIC-MS was optimized using 10 mM ammonium formate to reduce glycan peaks’ overlapping and minimize ionization suppression. Solvent A consisted of 10 mM ammonium formate in 40% acetonitrile/water (v/v), and solvent B was 90% acetonitrile/water (v/v) containing 10 mM ammonium formate. Both solvents were buffered to pH 4.0 with formic acid for the positive mode or buffered to pH 7.0 with ammonium for the negative mode. A rather flat gradient was delivered at 15 μl/min after a loading time of 25 min at 40°C: 10–17% A (25–45 min), 17–23%A (45–85 min), 23–30% A (85–105 min), and 30–80% A (105–145 min). Samples were reconstituted in 50 μl of 75% acetonitrile/water. The injection volume was 0.1 μl for all samples.

PGC separation was performed on a Hypercarb^TM^ KAPPA column (100 × 0.5 mm, 3 μm, Thermo Scientific) at a flow rate of 8 μl/min. PGC-MS was operated in the negative-ion mode, employing ammonium bicarbonate as the optimal mobile phase buffer. For each sample, 0.1 μl (dissolved in 50 μl of water) was loaded onto the analytical column and eluted with a binary solvent consisting of A (10 mM ammonium bicarbonate, pH 8.0) and B (acetonitrile) at 8 μl/min at 35°C. An optimized glycan gradient elution was used for the separation of glycan mixtures as follows: 2–10% B (0–20 min), 10%–16%B (20–110 min), and 16–30% B (110–150 min).

### Data Analysis

Raw LC/MS data were auto-processed into lists of neutral masses and abundances using the DeconTools software (Jaitly et al., 2009). GlycResoft combines the raw neutral mass peaks into compounds, correcting mass spectrometric adducts (Maxwell et al., 2012). The program scores the data, generates a list of candidate glycan compositions, and matches these against the compound list. Putative structures can be assigned based on known human milk oligosaccharide patterns: glycan compositions containing hexose (Hex), *N*-acetylhexosamine (HexNAc), fucose (Fuc), *N*-acetyneuraminic acid (NeuAc), the label group (2-AB, 2-AP, DAP, 2-AA, PMP, or the alditol form), and dehydration (–H_2_O) were considered. All glycan assignments were made within a specified tolerance level (≤5 ppm). Some of the data from the tandem MS analysis of the corresponding glycans were further processed using GlycoWorkbench to ensure correlation with database assignments. All the quantitative data were normalized to the total identified oligosaccharide peak area (in the format of percentage, %).

## Results and Discussion

**SCHEME 1:**
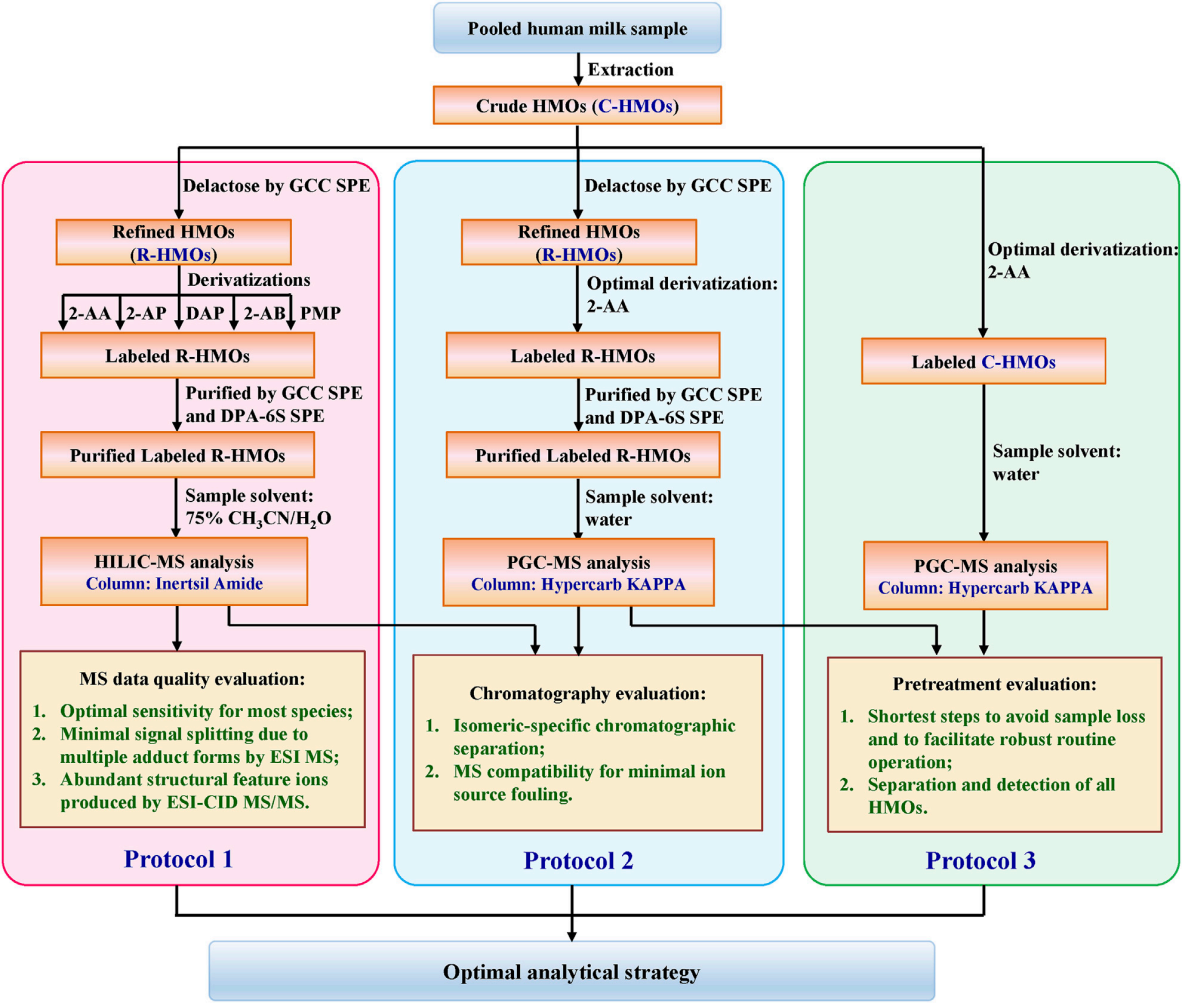
Schematic of the sample preparation process for comprehensive comparison.

The structural diversity and complexity of HMOs make the industry synthesis and production of various HMO standards very challenging. The limited available HMOs and their high cost made it difficult for us to start the assays by collecting a reasonable mixture of HMO standards in known relative proportions. To seek out an optimal strategy with broad applicability, a stock of HMOs pooled from 13 random healthy lactating mothers was instead used as our object since the person-to-person variability in glycosyltransferase expression affects the HMOs’ composition (Kobata, 1992; McGuire et al., 2017; Thurl et al., 2017). The overall workflow for comparing different combination strategies for HMO profiling is summarized in Scheme 1. Three protocols were developed sequentially to optimize HMO analysis. Protocol 1: lactose-free R-HMOs were derivatized by different labeling techniques, purified by SPE approaches, and analyzed by the HILIC-MS approach; Protocol 2: lactose-free R-HMOs were derivatized by the optimal labeling technique proposed, purified by SPE approaches, and analyzed by the PGC-MS approach; Protocol 3: raw C-HMOs with lactose were derivatized by the optimal labeling technique and analyzed by the PGC-MS approach directly. Chromatographic and MS data obtained from all the protocols are described below.

### Comparing Labeling Techniques in Terms of MS Data Quality

**FIGURE 1:**
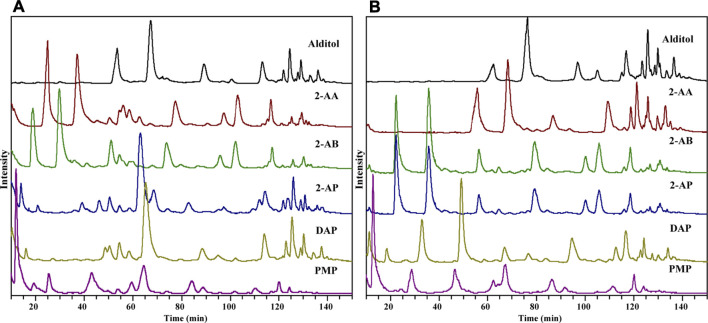
TIC of HMO derivatives by HILIC-MS in the positive-ion **(A)** and negative-ion **(B)** modes.

Six derivatization reactions were selected for HMO analysis in this comparative study (Supplementary Figure S1). 2-AB, 2-AP, DAP, 2-AA, or PMP labeling reactions were performed under strong acidic, weak acidic, or alkaline conditions as described in the experimental section. The reductive reaction of native glycans to glycan alditols was conducted under neutral conditions, acting as a reference method. With the design incorporating different reaction conditions in Protocol 1, LC-MS-based profiling analysis of HMOs utilizing different derivatizations could be systematically evaluated. Different LC separation and MS setting conditions will influence the ionization efficiency, the types of adducts formed, and the fragmentation patterns for differently derivatized oligosaccharides. To allow a fair comparison of different derivatized HMOs, a general HILIC was selected as our initial chromatography approach because it was usually performed well with all reductively tagged derivatives (Ahn et al., 2010; Lauber et al., 2015; Cesla et al., 2016). Also, to prevent MS signal contamination by salts, labeling reagents and lactose, a prelabeling GCC SPE, followed by a post-labeling DPA-6S SPE and GCC SPE, was employed to purify the analytes. The total ion chromatography (TIC) of HMOs derivatized by 2-AA, 2-AB, 2-AP, DAP, and PMP is shown in Figure 1, with reduced HMO alditols as a reference. To compare the compatibility of each derivatization with the MS detection, we looked into their ionization behaviors in ESI-MS, their fragmentation patterns in CID-MS/MS, their compositional profiling results and their relative sensitivity based on the MS signal strength.

#### Ionization Behaviors of Different Derivatives

HMOs can be classified into four groups according to their residual modification: fucosylated neutral HMOs, nonfucosylated neutral HMOs, sialylated acidic HMOs, and the fucosylated and sialylated acidic HMOs. To avoid biased comparison, four glycan compositions were selected as representatives of the four HMO classes. The representative glycan compositions (denoted numerically in this text as Fuc-Hex-HexNAc-Neu5Ac; nomenclature is described in Supplementary Table S1) were as follows: 1-2-0-0 (FL series, for fucosylated neutral HMOs), 0-3-1-0 (LNT series, for nonfucosylated neutral HMOs), 0-3-1-1 (LST series, for sialylated acidic HMOs), and 1-4-2-1 (MFMSLNH series, for fucosylated and sialylated acidic HMOs).

In the positive ESI-MS mode, all five labeled derivatives formed consistent abundant protonated ions ([M + H]^+^ or [M+2H]^2+^) for four representative glycans (Supplementary Figure S2; Supplementary Table S2), while the reference alditol derivatives produced heterogeneous sodium adduct ions ([M + H]^+^, [M+2H]^2+^, [M + Na]^+^, and [M + NH_4_]^+^) and exhibited inconsistent ionization patterns between fucosylated HMOs (Supplementary Figures S2A,D) and nonfucosylated HMOs (Supplementary Figures S2B,C). In the negative mode, 2-AB, 2-AP, and DAP derivatives unexpectedly produced heterogeneous and inconsistent molecular ions for four glycans ([M–H]^–^, [M–2H]^2–^, [M + HCOO]^–^, [M + HCOO–H]^2–^, [M+2HCOO + Na]^–^, [M+2HCOO–H + Na]^2–^, [M + HCOONH_4_–H]^–^, [M + HCOO–H + Na]^–^, and [M + Na–2H]^–^) (Supplementary Figure S2) as well as the reference alditols. On the contrary, 2-AA and PMP derivatives still shared a consistent ionization pattern by prominent deprotonated ions ([M–H]^–^ or [M–2H]^2–^) for all representative glycans. The less the sodium adducts formed for each HMO, the more promising the labeling technique is since this can help solve the notorious problem of unfair detection of hetero-oligosaccharides caused by inconsistent molecular ion signal splitting.

#### Fragmentation Patterns of Different Derivatives

2′-FL of the FL series (1-2-0-0), LNT of the LNT series (0-3-1-0), LST-b of the LST series (0-3-1-1), and F-LST-a of the MFMSLNT series (1-3-1-1) were further selected as representatives for comparison analysis of their fragmentation efficiency. Their MS/MS spectra were characterized (Supplementary Figure S3; Supplementary Table S3) using the systematic nomenclature for carbohydrate fragmentation (Domon and Costello, 1988), assisted with the fragmentation rules reported previously (Lang et al., 2014; Liu et al., 2015; Lang et al., 2018).

All six positive ESI-CID MS/MS spectra of 2′-FL derivatives were characterized by exclusive Y-type cleavage at every glycosidic bond (Supplementary Figure S3A). Four LNT derivatives (2-AP, 2-AB, DAP, and PMP) characterized by abundant Y ions; one LNT-2-AA derivative produced comparable abundance of Y ions and B_2_ ions, and the reference LNT-alditol was fragmented into prominent B ions and weak Y ions instead (Supplementary Figure S3B). For sialylated acidic HMO, LST-b, three derivatives (2-AP, DAP, and PMP) produced only trace amounts of Y ions and the reference alditol did not produce any Y ions, while 2-AA and 2-AB derivatives were featured by abundant diagnostic glycosidic cleavage ions with a balanced intensity distribution, for example, Y ions, B ions, specific D ions, and characteristic Y/Y-type ions (Supplementary Figure S3C). For the longer acidic F-LST-a, unfortunately, the positive-ion mode did not provide any informative CID-MS/MS fragmentation spectra for all six derivatives, probably due to the big molecular size and the negatively charged sialic acid group.

Under negative-ion conditions (Supplementary Figure S3), the fragmentation varied substantially for four representative glycans for three derivatives (2-AB, 2-AP, and DAP) and the reference alditols, such as intense cross-ring ions (^1,3^A_2_ ions) for 2′-FL, extensive glycosidic cleavages (Y_2_, Z_3_, and B_2_ ions) for LNT, or minor fragment ions (Y_3β_, ^0,2^X_3β_ ions) for acidic LST-b and longer acidic F-LST-a. PMP derivatives gave the least satisfactory identification spectra for all four glycans because the loss of PMP from [M–H]^–^ and Z_1_ ions competitively inhibited the production of other diagnostic sugar cleavage ions. Surprisingly, 2-AA derivatives produced sequentially fragmented Y ions (e.g., Y_1_, Y_2_, Y_3_, etc.) with high abundance for all four representatives. This consistent fragmentation information for a mixture of hetero-oligosaccharides could work as a reference for future research on optimizing the multiple reaction monitoring-mass spectrometry (MRM-MS) technique for a wide-range detection of HMOs in biological mixtures which usually have significant structure and size distribution (Fong et al., 2011; Mank et al., 2019).

#### Compositional Analysis of HMOs by Different Labeling Techniques

**FIGURE 2:**
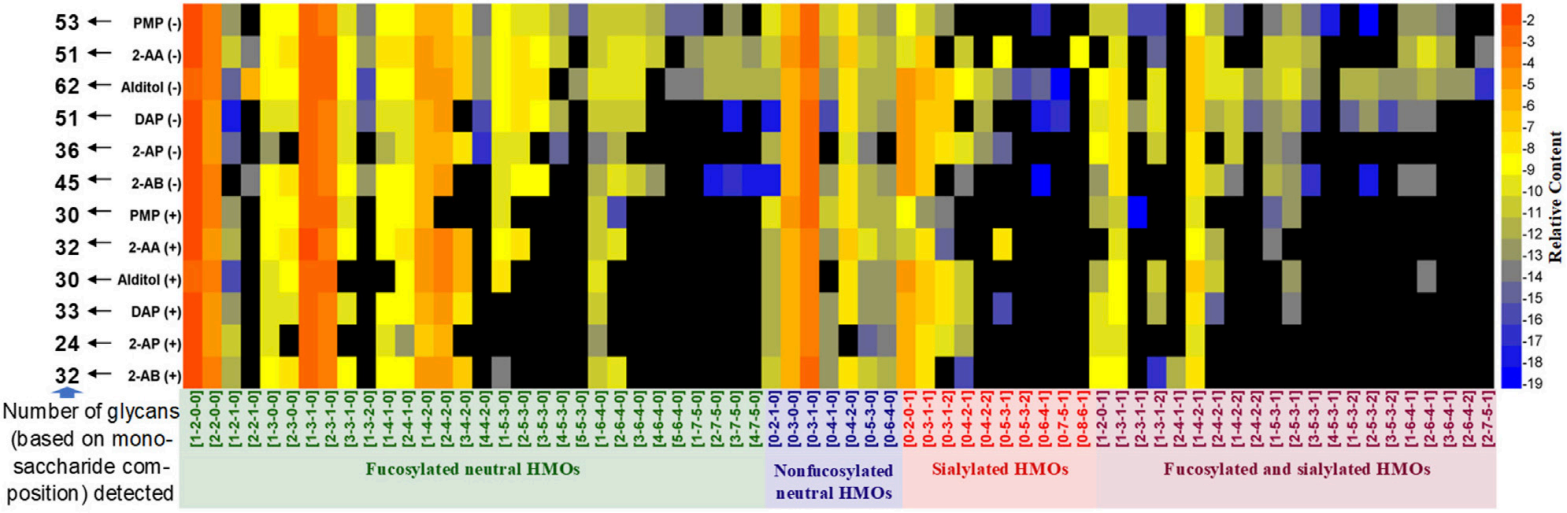
Relative quantifications of all HMO peaks for 2-AB, 2-AP, DAP, alditol, 2-AA, and PMP derivatives in the positive (+) and negative (−) modes depicted as a heatmap. In the heatmaps, the region in black indicates no detectable glycans, and glycans are marked with four colors based on the classification of structure features.

Following data acquisition on HILIC-MS, peaks were assigned based on the monosaccharide composition (Fuc-Hex-HexNAc-Neu5Ac, x-x-x-x) (Supplementary Figure S4). Retention of the four HMO classes can be easily observed: fucosylated neutral (blue), nonfucosylated neutral (green), sialylated acidic (pink), and fucosylated and sialylated acidic (red). The overlaid extracted glycan chromatograms illustrate the extensive and complex glycan components. Total HMO intensities for each derivative were normalized to 100% to allow compositional profiling analysis. A clear view of the composition distribution was easily discerned when the numeric data of relative quantities of the glycan structures were further normalized by logarithmic (base 2) and transformed in a heatmap (Figure 2).

The total number of glycan compositions (peaks of isomers were summed as one glycan composition) identified for all derivatives was 68. As expected, for all six types of derivatives, the negative ESI-MS mode rendered 12–32 more HMOs (monosaccharide compositions) detectable than the positive-ion mode did. More minor sialylated acidic HMOs (e.g., 0-4-2-**2**, 0-5-3-**2**, 0-6-4-**1**, 0-7-5-**1**, 0-8-6-**1**, 1-4-2-**2**, 2-4-2-**2**, 3-5-3-**1**, 4-5-3-**1**, 1-5-3-**2**, 2-5-3-**2**, 3-5-3-**2**, 1-6-4-**1**, 3-6-4-**1**, 2-6-4-**2**, and 2-7-5-**1**) and minor fucosylated neutral HMOs with large molecular weights (e.g., **4**-4-2-**0**, **3**-5-3-**0**, **4**-5-3-**0**, **5**-5-3-**0**, **3**-6-4-**0**, **4**-6-4-**0**, **5**-6-4-**0**, **1**-7-5-**0**, **2**-7-5-**0**, **3**-7-5-**0**, and **4**-7-5-**0**) were detected under negative HILIC-MS conditions. In addition, the reference alditol strategy (reduced but not labeled) enabled the most glycans (62 monosaccharide compositions) being detected; the labeling strategies of PMP, 2-AA, and DAP allowed the moderate numbers (51–53 monosaccharide compositions) of glycans detected, while the labeling strategies of 2-AB and 2-AP allowed less than 45 numbers of glycans (monosaccharide compositions) detected. Therefore, for qualification analysis, especially the in-depth structural characterization of a mixture of HMOs, reducing the oligosaccharides to their alditols and analyzing in the negative-ion MS mode would benefit the analysis best by covering the most structures.

Previously published work from the Lebrilla group using the chemical reduction and PGC-TOF-MS characterized the fine structures of 45 neutral glycans (isomers) (18 monosaccharide compositions) and 30 sialylated acidic glycans (isomers) (14 monosaccharide compositions), establishing very fruitful libraries for HMO structures (Wu et al., 2010; Wu et al., 2011). With the milk oligosaccharide standards available, the absolute quantification methods for measuring the predominant HMOs were further developed by them using MRM (Hong et al., 2014). As discussed above in *Ionization Behaviors of Different Derivatives* and *Fragmentation Patterns of Different Derivatives*, the reference alditols produced heterogeneous adduct molecular ions for four representative glycans in the negative-ion mode, which may lead to the unfair detection of hetero-oligosaccharides for ESI-MS1-based profiling analysis, and the fragmentation patterns varying substantially between glycans, which theoretically might increase the sensitivity difference between different glycans when applied for the MRM-based absolute quantification analysis. To figure out whether the former factor will affect the profiling analysis of HMOs, we compared the sensitivity of six types of derivatives based on MS1 signal intensity.

#### Relative Sensitivity Evaluation for Different Labeling Techniques

**FIGURE 3:**
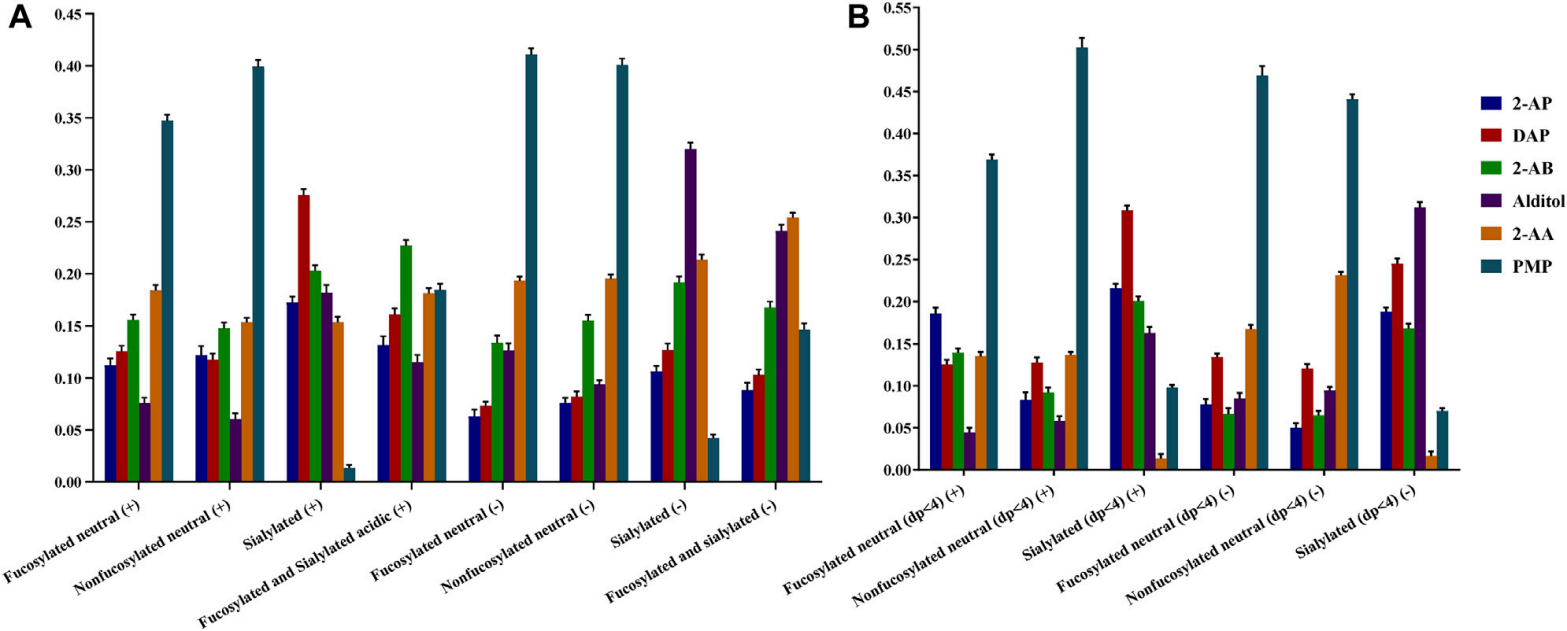
Comparison of ESI-MS ionic abundances observed for 2-AB, 2-AP, DAP, alditol, 2-AA, and PMP derivatives in the positive (+) and negative (−) modes. Data acquired from common identified glycan peaks comprising four or more than four monosaccharides **(A)** and trisaccharides **(B)**. For each type of derivative, three reactions were set up, and three measurements were taken on each product. Percent errors were on the order of ±5%.

To achieve the sensitivity comparison analysis of six derivatives, the signal intensities of common identified glycans for each HMO class were summed for each derivative (Figure 3). Since several papers have reported the specific loss of 3-FL during the clean-up step of GCC-SPE (Blank et al., 2011; Xu et al., 2017; van Leeuwen, 2019), we split the comparison into two sessions: HMOs with more than four monosaccharides (Figure 3A) and HMOs with three monosaccharides (Figure 3B).

For comparison of the HMOs with more than four monosaccharides (Figure 3A), the order of detection sensitivity for 2-AB, 2-AP, DAP, and alditol derivatives varied substantially for four HMO classes in both ion modes, which agreed with our hypothesis that the formation of heterogeneous sodium adduct ions would split the signal of each peak and thus lead to a variation of sensitivity between different glycans. Therefore, we expected enhanced sensitivity by 2-AA or PMP derivatizations, which promises consistent ionization behaviors for different HMO classes. The intensities of 2-AA and PMP derivatives did rank the second highest or the highest for two neutral HMO classes in both ion modes. For the sialylated HMO class in positive, the signal of PMP derivatives, however, ranked the lowest, while the signal of 2-AA derivatives was comparable to that of 2-AP derivatives, which was not satisfactory. For two acidic HMO classes in the negative-ion mode, despite the low intensities of PMP derivatives, the intensities of 2-AA derivatives ranked the second highest for sialylated HMOs and even the highest for fucosylated and sialylated HMOs. The overall enhanced sensitivity by 2-AA derivatization for all four HMO classes made 2-AA more promising. This observation of sensitivity difference between different structural featured HMOs indirectly proved that the varied multiple adduct sodium forms will affect the glycan profiling results.

Another comparison on the small trisaccharides (Figure 3B) however revealed that 2-AA derivatization had an additional specific loss of sialylated lactose (SL) series (0-2-0-1) compared with other labeling methods. The SL series are the most abundant components of acidic HMOs and have become a hot spot in HMO biology research (Weiss and Hennet, 2012; Kurakevich et al., 2013; Moon et al., 2016; Hobbs et al., 2021).

For preventing the loss of 3-FL, Xu et al. skipped the SPE step and directly injected the reduced HMO alditols containing an overload of lactose into LC-MS; however, 2′-FL was co-eluted with the abundant lactose, and thus, its ion was suppressed by lactose (Xu et al., 2017). Subsequently, Gu et al. developed an approach to quantitate HMOs including 3-FL by combining three analytical methods, HPAEC-PAD (for determining 3-FL), PGC-LC-MS (for other HMOs), and one-dimensional ^1^H-NMR (for showing relative levels of different structural elements), following a GCC-SPE process (Gu et al., 2021a). This approach provided more accurate information on the relevant HMOs, although the operation process was labor-intensive, thus posing challenges to the development of high-throughput protocols (Gu et al., 2021a). Here, we also expect to optimize the 2-AA-associated analytical protocols further to avoid the selective loss of small HMOs since 2-AA labeling had shown quite beneficial observations for most of the medium or large HMOs.

### Comparing Three Combination Strategies in Terms of Pretreatment and LC-MS Methods

To achieve a robust glycan profiling analysis, we seek to develop a compatible LC-MS system which can separate and determine the HMOs with reasonable resolution as well as suppress the interference from excess salt, labels, and lactose. As is known, reversed-phase chromatography (RPC) is considered a mature technique that can separate the ionic salts, less polar analytes, and hydrophobic molecules reasonably based on their polarity level difference. For our derivatized HMOs, the hydrophobic label group does prefer to bind to the RPC stationary phase, whereas the extremely polar glycan moiety tends to be washed out with extremely weak retention. Since the fluorescent label hydrophobic moiety is much smaller than the glycan hydrophilic moiety, the overall retention of 2-AA-labeled HMOs is very weak on the conventional RPC. Alternatively, PGC, which has been called the ultimate reversed-phase material, shows stronger adsorption of polar analytes. PGC has been employed successively for glycomic analysis of reduced milk oligosaccharide alditols (Wu et al., 2010; Wu et al., 2011). Inspired by Xu’s SPE skipping process and Wu’s PGC-based glycomic analysis work, we optimized our 2-AA labeling-based HMO profiling analysis by designing another two analytical protocols → SPE-labeling-SPE-PGC/MS (referred to as Protocol 2) and labeling-PGC/MS (referred to as Protocol 3), in addition to SPE-labeling-SPE-HILIC/MS described in sections above (referred to as Protocol 1) (Scheme 1). Protocol 2 used the same sample loaded to Protocol 1, meaning that it was carried out with the same SPE route as Protocol 1; this aims to seek out whether an enhanced LC separation capacity or a similar level of MS data quality could be acquired with PGC-MS, compared with HILIC-MS. Protocol 3 cut off all the cleanup SPE steps that were utilized in Protocol 1 and Protocol 2; this aims to seek out whether the selective loss of specific HMOs could be prevented with the simple rapid sample preparation process.

#### Evaluation of the Chromatographic Separation and Mass Spectrometric Data Quality

**FIGURE 4:**
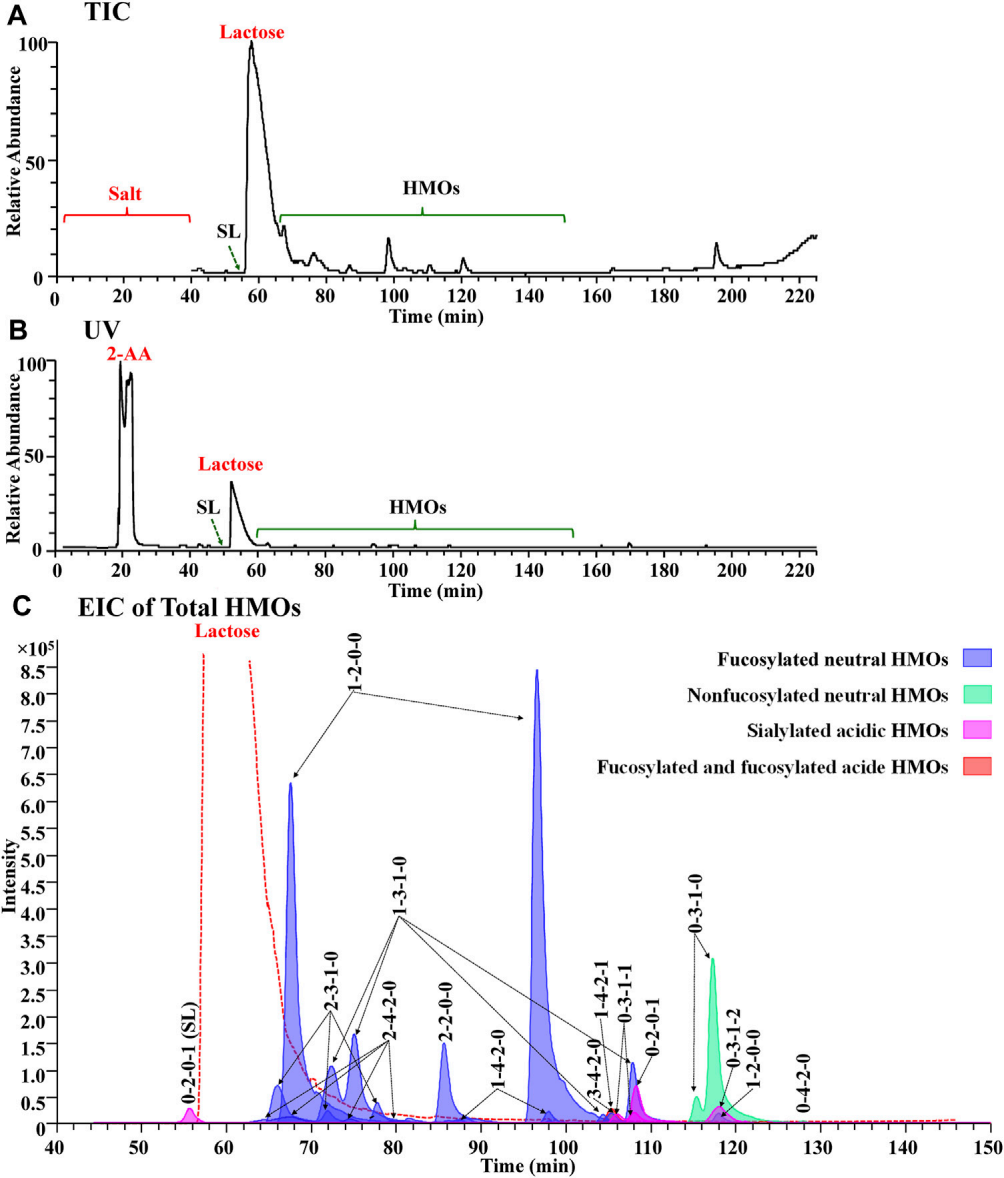
Full MS compatibility of the PGC method in the Protocol 3 strategy was demonstrated by the agreement of TIC **(A)**, UV **(B)**, and EIC of total HMOs **(C)**. In **(C)**, each glycan class was designated with a different color: fucosylated neutral (blue), nonfucosylated neutral (green), sialylated acidic (pink), and fucosylated and sialylated acidic (red).

In the preliminary part of this work, a series of experimental PGC-MS conditions were optimized to achieve a satisfactory LC separation and noncontamination compatibility with MS. Using a buffer system of 10 mM ammonia bicarbonate for both Protocol 2 and Protocol 3, we obtained a surprisingly good chromatogram from Protocol 3, with online removal of salts and labels from total milk carbohydrates, very limited overlapping between labeled lactose and labeled HMOs, and resolute separation of most labeled HMOs (Figure 4). The ionic salts and the hydrophobic 2-AA labels were co-washed out 10 minutes away from HMOs, allowing us to use the diverter valve built in an LTQ-Orbitrap XL mass spectrometer to automatically switch the contaminating eluents to the waste (Figures 4A,B). The retention of the overload 2-AA-labeled lactose between that of 2-AA-labeled SL (0-2-0-1) and that of the other 2-AA labeled HMOs (Figure 4C) prevented the signal suppression from lactose to the HMOs, making both 2-AA labeling and the SPE-skipping process unique for the PGC-MS method.

**FIGURE 5:**
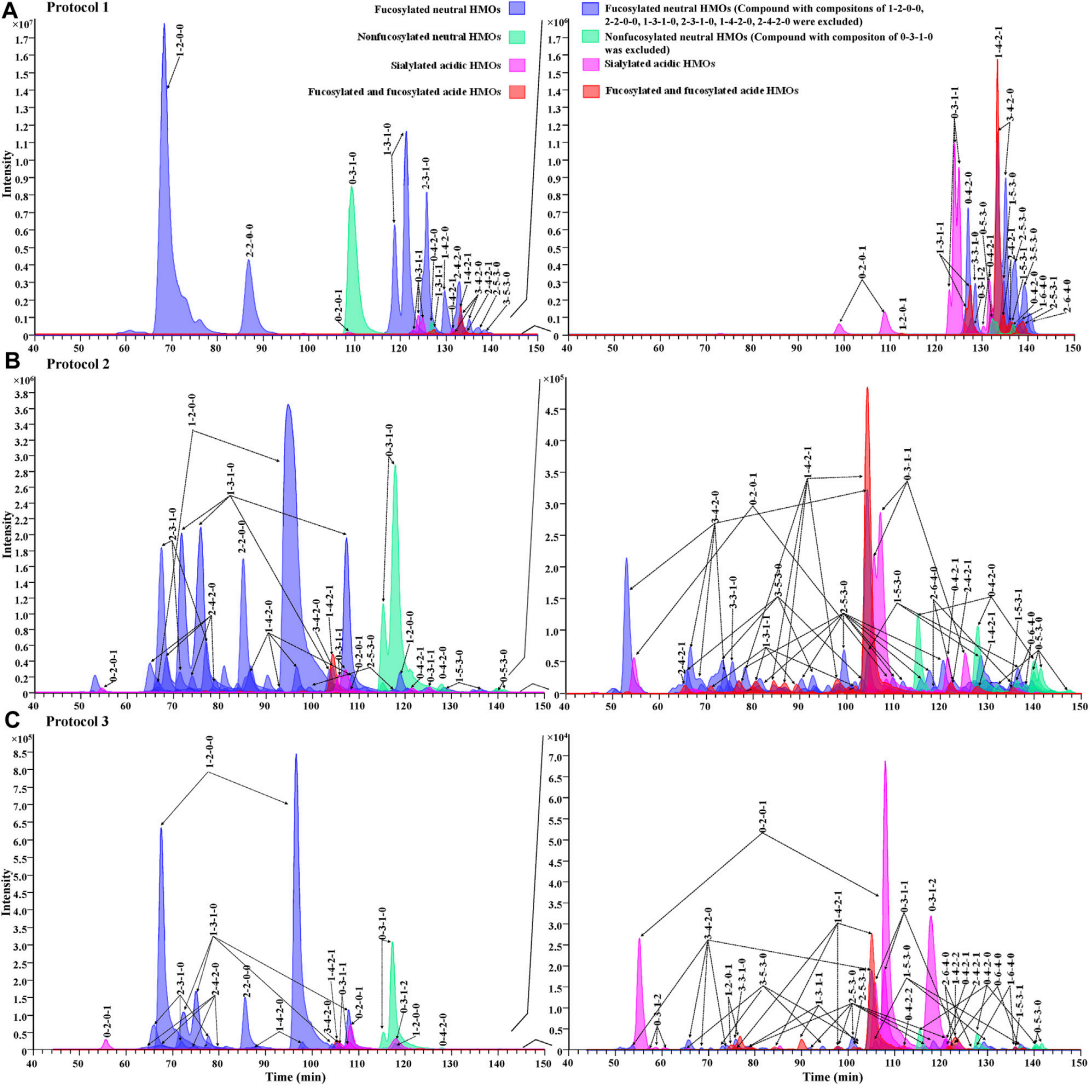
Overlaid EIC showing the elution profile of 2-AA-labeled HMOs via Protocol 1 **(A)**, Protocol 2 **(B)**, and Protocol 3 **(C)** in the negative mode. Each glycan class was designated with a different color: fucosylated neutral (blue), nonfucosylated neutral (green), sialylated acidic (pink), and fucosylated and sialylated acidic (red). Figures on the right side of the panels show low-abundance peaks (compositions of major peaks, including x-2-0-0, x-3-1-0, x-4-2-0, 0-3-0-0, and 0-3-1-0, were excluded, and x = any number of fucose residues).

For the evaluation of chromatographic separation of 2-AA-labeled HMOs, we compared the chromatograms obtained from Protocol 1, Protocol 2, and Protocol 3 (Figure 5) and observed that the retention behaviors of 2-AA-labeled HMOs differed on two chromatography modes. The HILIC method (Protocol 1) was somewhat referred to as the “size separation” method because the retention correlated to the size of HMOs (Figure 5A). In contrast, the PGC method (Protocol 2 and Protocol 3) offered remarkable chromatographic separation of most glycans (Figures 5B,C) based on a combination mechanism of size, charge, and conformation properties. Supplementary Table S4 lists the counts of visible extracted ion chromatogram (EIC) peaks for each monosaccharide composition obtained from the three protocols. Using the same sample preparation process, Protocol 1 provided 32 visible EIC glycan peaks (for 23 monosaccharide compositions), while Protocol 2 increased the visible EIC separation to 74 glycan peaks (for 23 monosaccharide compositions), indicating the powerful separation of isomers by PGC. Using the same PGC-MS method as Protocol 2, Protocol 3 had 74 EIC glycan peaks visibly determined based on 29 monosaccharide compositions. Protocol 3 had six more monosaccharide compositions extractable (e.g., 1-2-0-1, 0-3-1-2, 0-4-2-2, 1-4-2-2, 2-5-3-1, and 1-6-4-0), suggesting that the simple rapid sample preparation avoided selective loss of these glycans. The reason why Protocol 3 had more monosaccharide compositions extracted did not have more visible EIC peaks (counting the isomers) than Protocol 2 might rely on the fact that the amount of HMOs injected in Protocol 3 is about 10-fold less because of the high ratio of lactose and some minor isomeric peaks were too low to be visibly observed. Increasing the injection amount of the analytes in further future studies will improve the number of glycans determined. Between Protocols 2 and 3, there was little retention time shifted or little chromatographic resolution decreased, indicating that the injected interferences associated with Protocol 3 did not affect the retention capacity of 2-AA-labeled HMOs on the PGC column.

As described in *Compositional Analysis of HMOs by Different Labeling Techniques*, Wu et al. characterized 45 neutral glycans (isomers) (18 monosaccharide compositions) (Wu et al., 2010) and 30 sialylated acidic glycans (isomers) (14 monosaccharide compositions) (Wu et al., 2011) in 2 separate famous works. The elegancy of the Protocol 3 approach is that it allowed a comparable number of glycan peaks (74) determined without fractioning the HMOs into neutral and acid parts. With further detailed characterization of ESI-CID-MS/MS spectra, an alternative library of 2-AA-labeled HMOs could be established in addition to the existing libraries of reduced HMO alditols. Considering this study focused on the compositional profiling analysis of HMOs based on monosaccharide constitution, the full identification of each HMO isomer for the Protocol 3 strategy is not described in this context. With each visible EIC peak representing an HMO structure, it has the potential to allow 74 HMOs semi-quantified or absolute-quantified when standards are available (Austin et al., 2016; Austin and Bénet, 2018; Austin et al., 2019; Samuel et al., 2019; Gu et al., 2021b). Further validation work by the MRM approach is warranted for this point (Hong et al., 2014; Totten et al., 2014; Xu et al., 2017).

Moreover, the ionization behaviors for four representative HMOs in PGC-MS (Protocol 2 and Protocol 3) were consistent (Supplementary Figure S5) to produce predominant deprotonated molecular ions in the negative-ion mode, providing the same MS data quality as HILIC-MS (Protocol 1). A comprehensive glycan profile can therefore be obtained with fair detection sensitivity of each structural element. Using the logarithm embedded in GlycResoft, we can analyze the relative abundance of each monosaccharide compositions in a high-throughput way, with much better sensitivity and accuracy than the manual integration of each EIC peak area.

#### Evaluation of the Sample Loss With or Without Pretreatment

**FIGURE 6:**
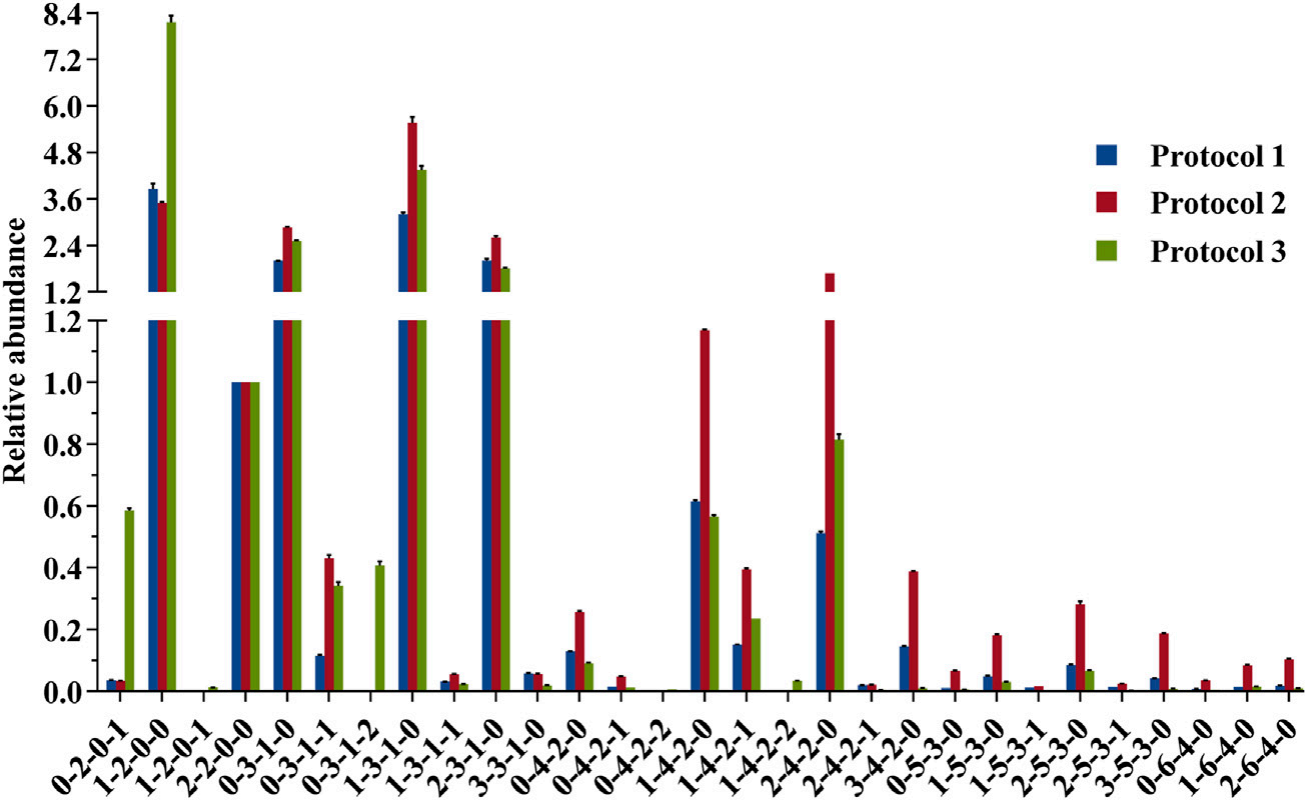
Comparison of the relative abundances of 2-AA-labeled HMOs derived from 3 protocol strategies in the negative mode. Data were acquired from common major glycan peaks. LDFT (2-2-0-0) was selected as the internal standard element for a fair comparison. For each strategy, three measurements were taken on each product. Percent errors were on the order of ±5%.

The glycan profile for human milk using each protocol was characterized based on monosaccharide composition. To achieve a fair comparison between the three protocols, we need to select a reasonable internal glycan to normalize the relative abundances. This internal glycan for normalization analysis should not be involved in selective loss of small or large HMOs associated with SPE, liable to loss of sialic acid associated with the ESI source, or the signal ambiguated by isomeric separation. LDFT, a neutral difucosyllactose tetrasaccharide with only one structural configuration [Fucα1-2Galβ1-4(Fucα1-3) Glc-label], was therefore selected as the optimal internal standard for the comparative profiling analysis (Figure 6).

Comparing the glycan profiles acquired from HILIC-MS and PGC-MS using the same sample, Protocol 2 gave higher abundance ratios of larger glycans (e.g., x-3-1-x, x-4-2-x x-5-3-x, and x-6-4-x series) and lower abundance ratios of small glycans than Protocol 1 did. A possible explanation for this result may rely on the fact that the HILIC system needs a starting eluent of a high acetonitrile content (75%), requesting a similar sample solution before injection, thus resulting in possible precipitation of the large glycans (Ruhaak et al., 2010), while PGC needs an aqueous solvent as the starting eluent, making water as the sample solution, thus allowing all polar glycans loaded to the system. Another explanation for the ratio distribution of small HMOs and large HMOs may be the low recovery of the internal standard LDFT on the PGC column specifically, but it is hard to believe that the recovery of other larger neutral HMOs was improved while this medium tetrasaccharide was not. The elution position of LDFT in HILIC was about 86 min and in PGC was about 85 min, with almost no interferences happening at this elution position. As published previously by Austin et al., using HILIC and 75% acetonitrile as the sample loading solution, they obtained recoveries of spiked standards between 85 and 120% for all structures tested, including some quite large glycans (Austin and Bénet, 2018), which is also true. Since their calibration study was carried out on one system, the solubility issue might contribute to the systematic error only. This ratio distribution difference of small and large HMOs between two LC-MS systems indicates that the compositional profiling analysis of HMOs can be influenced by the two different LC-MS systems, but this does not mean that bias would happen for the standard-calibration-based quantification study via one LC-MS system. Nevertheless, further tests will be needed for the solubility/precipitation-associated loss of HMOs in 75% acetonitrile.

**FIGURE 7:**
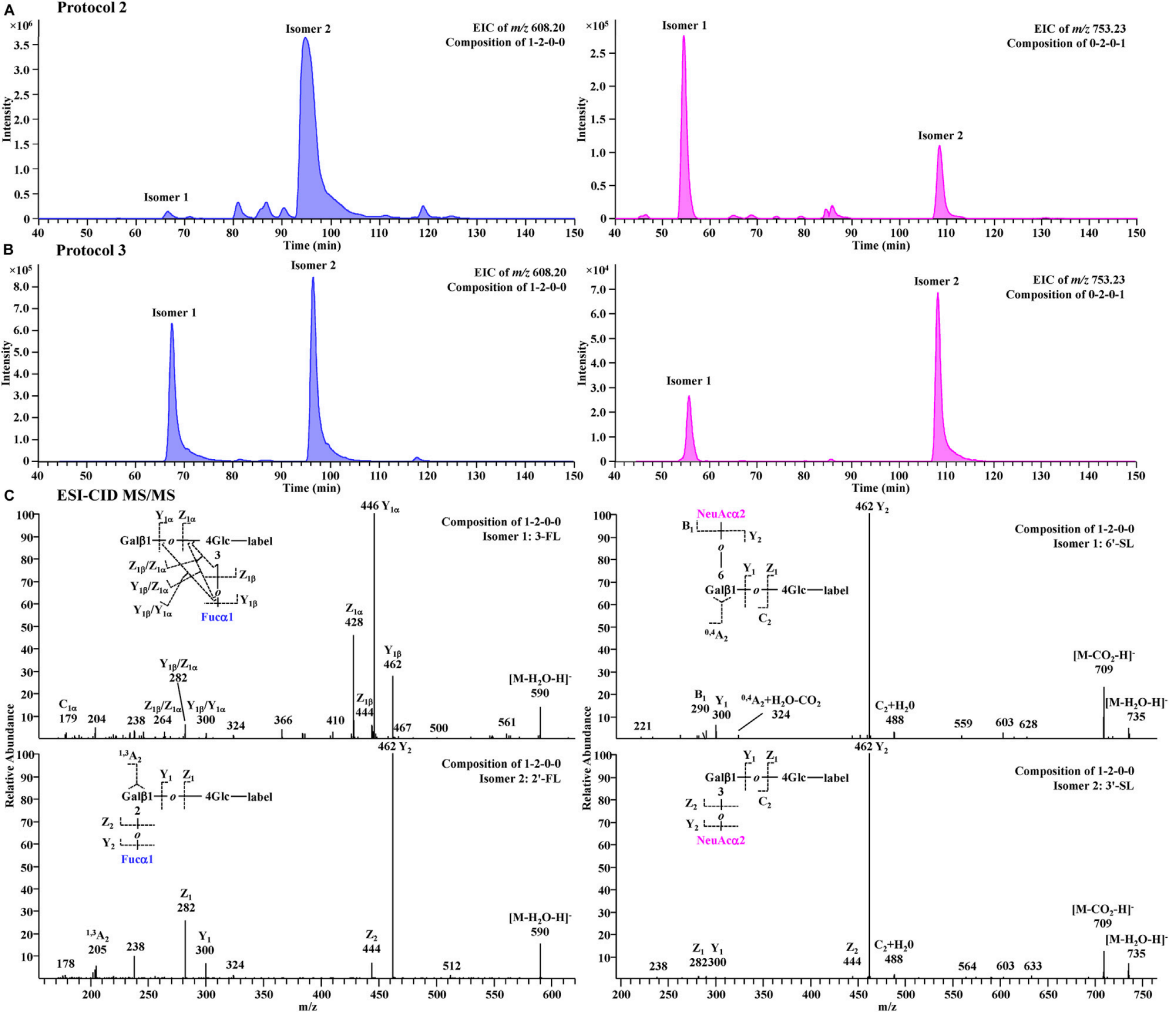
Extracted chromatograms for FL series (m/z 608.20) and SL series (m/z 753.23) in Protocol 2 **(A)** and Protocol 3 **(B)** and their CID-MS/MS spectra **(C)**.

A further comparison of glycan profiles acquired from the same PGC-MS with or without SPE approaches indicated that Protocol 3 provided higher abundance ratios for most small glycans, especially the FL series and SL series, than Protocol 2. For example, glycan species 1-2-0-0 (FL series) showed an ∼2-fold higher abundance ratio in Protocol 3 profiles than in Protocol 2, and glycan species 0-2-0-1 (SL series) showed an ∼17-fold higher abundance ratio in Protocol 3 than in Protocol 2. To identify which glycan isomers were selectively lost for FL and SL series during the SPE process, we extracted their ion chromatograms (EIC) from Protocol 2 and Protocol 3 (Figures 7A,B). Detailed structures of the isomeric peaks regarding linkage positions were characterized (Figure 7C) according to the tandem mass spectra fragmentation principles reported previously (Wheeler and Harvey, 2000; Chai et al., 2001; Chai et al., 2002). 3-FL [Galβ1-4(Fucα1-3)Glc-label], one of the two FL isomers, was determined by the glycosidic cleavages, C_1α_ (*m/z* 179), Y_1α_ (*m/z* 446), and Z_1α_ (*m/z* 428). For the other isomer, 2′-FL (Fucα1-2Gal β1-4Glc-label), the cross-ring cleavage, ^1,3^A_2_ ion at *m/z* 205, clearly indicated that the glycosidic bond of Fuc to Gal was (1→2). The isomer of SL series, 6′-SL (NeuAcα1-6Galβ1-4Glc-label), was also distinguished from 3′-SL (NeuAcα1-3Galβ1-4Glc-label) by the cross-ring cleavage, ^0,4^A_2_+H_2_O-CO_2_ ion (*m/z* 324). Normalized by LDFT, we compared their relative EIC peak area abundances between Protocol 2 and Protocol 3 (Supplementary Figure S6). While 2′-FL showed comparable abundances between two protocols, the relative abundance of 3-FL was ∼60-fold higher in Protocol 3 than in Protocol 2. For SL isomers, Protocol 3 provided ∼6 and ∼40-fold higher relative abundances for 6′-SL and 3′-SL (>5 fold) than Protocol 2. The issue of selective sample loss of trisaccharides, especially 3-FL and 3′-SL, associated with the SPE approaches was therefore confirmed for both Protocol 1 and Protocol 2. In addition to the selective loss of 3-FL reported previously, the loss of 3′-SL in these SPE steps should be paid attention to. Protocol 3 prevents such sample loss extensively. In addition, for the disialylated acidic glycan species (e.g., 0-3-1-2, 0-4-2-2, and 1-4-2-2), Protocol 3 rendered them much more detectable than the other two protocols, reducing the loss of two negative charged glycan series. These observations proved Protocol 3 an adequate and reliable profiling analysis.

## Conclusion

This study evaluated the influence of glycan labeling, sample preparation, and LC-MS methods on the compositional profiling analysis of HMOs. With an identical MS data quality, a robust chromatographic separation, and a rapid simple sample preparation, an analytical protocol of 2-AA labeling, followed by a direct PGC-MS analysis, provided an enhanced profiling analysis of HMOs. Benefited by the simple sample preparation process, 96-well plates can be employed to enable the high-throughput analysis in future studies. With the sensitivity improved nonselectively, this analytical protocol is further expected to increase the number of HMOs quantifiable in a natural biological mixture by integrating with MRM-MS (Yuan et al., 2005; Unterieser and Mischnick, 2011). Full in-depth sequence characterization and isobaric glycan distinguishability are further needed to update the glycomic library based on this analytical strategy. This research will provide more tools and methods to bring the science of human milk oligosaccharides forward.

## Data Availability

The original contributions presented in the study are included in the article/Supplementary Material, and further inquiries can be directed to the corresponding authors.
