# Supplementary material for: Comparison of Different Labeling Techniques for the LC-MS Profiling of Human Milk Oligosaccharides
Source: Front Chem. 2021 Sep 13;9:691299. doi: 10.3389/fchem.2021.691299 (PMC8473617; doi:10.3389/fchem.2021.691299)
Supplement: Supplementary file 1 [file DataSheet1.docx]

Comparison of Five Labeling Techniques for the LC-MS Profiling of Human Milk Oligosaccharides

Supplementary Material

# CONTENTS

Table S1. Structure of the main human milk oligosaccharides.

Table S2. The major molecular ions for FL, LNT, LST and MFMSLNH derivatives of 2-AB, 2-AP, DAP, alditol, 2-AA and PMP produced from positive and negative ESI-MS.

Table S3. The predominant fragment patterns for 2'-FL, LNT, LST-b and F-LST-a derivatives of 2-AB, 2-AP, DAP, alditol, 2-AA and PMP produced from positive and negative ESI-CID-MS/MS.

Figure S1. Derivatizations of HMOs. (**A**) 2-AA, 2-AB, 2-AP and DAP labeling via reductive amination; (**B**) PMP labeling via a Michael-type addition; and (**C**) reduction of HMOs into their alditols.

Figure S2. Positive-ion (+) and negative-ion (-) ESI-MS spectra of FL (**A**), LNT (**B**), LST (**C**) and MFMSLNH (**D**) derivatives of 2-AB, 2-AP, DAP, alditol, 2-AA and PMP.

Figure S3. Positive-ion (+) and negative-ion (-) ESI-CID MS/MS spectra of 2'-FL (**A**), LNT (**B**), LST-b (**C**) and F-LST-a (**D**) detected in the reduced alditol form and derivatized with 2-AB, 2-AP, DAP, 2-AA and PMP.

Figure S4. Overlaid extracted compound chromatogram (ECC) showing the elution profile of derivatized HMOs via HILIC-MS in positive (**A**) and negative (**B**) mode.

Figure S5. Negative-ion (-) ESI-MS spectra of FL (**A**), LNT (**B**), LST (**C**) and MFMSLNH (**D**) derivatives of 2-AA in protocol 1, protocol 2 and protocol 3.

Figure S6. (**A**) Comparison of the relative abundance of FL isomers and SL isomers in protocol 2 and protocol 3 (LDFT was selected as the internal standard); (**B**) Comparison of isomeric ratio of 3-FL/2ꞌ-FL and 6ꞌ-SL/3ꞌ-SL in protocol 2 and protocol 3.

# Supplementary Figures and Tables

**Table S1**. Structure of the main human milk oligosaccharides.

| Glycan |  | Detailed Structure | Nomenclature in this paper (Fuc-Hex-HexNAc-NeuAc) |
| --- | --- | --- | --- |
| LNDFH II | Lacto-N-difuco-hexaose II | Galβ1-3(Fucα1-2)GalNAcβ1-3Galβ1-4(Fucα1-2)Glc | 2-3-1-0 |
| DFLNH | Difucosyl-lacto-N-hexaose | Galβ1-4(Fucα1-3)GlcNAcβ1-6[Galβ1-3(Fucα1-4)GlcNAcβ1-3]Galβ1-4Glc | 2-4-2-0 |
| DFLNH I | Difucosyl-lacto-N-hexaose I | Fucα1-2Galβ1-3(Fucα1-4)GlcNAcβ1-3Galβ1-4Glc | 2-3-1-0 |
| 3-FL | Fucosyl-lactose | Galβ1-4(Fucα1-3)Glc | 1-2-0-0 |
| LNFP III | Lacto-N-fucopentaose III | Galβ1-4(Fucα1-3)GlcNAcβ1-3Galβ1-4Glc | 1-3-1-0 |
| LNFP II | Lacto-N-fucopentaose II | Galβ1-3(Fucα1-4)GlcNAcβ1-3Galβ1-4Glc | 1-3-1-0 |
| LDFT | Lacto-difuco-tetraose | Fucα1-2Galβ1-4(Fucα1-3)Glc | 2-2-0-0 |
| 2ꞌ-FL | 2ꞌ-Fucosyl-lactose | Fucα1-2Galβ1-4Glc | 1-2-0-0 |
| LNFP I | Lacto-N-fucopentaose I | Fucα1-2Galβ1-3GlcNAcβ1-3Galβ1-4Glc | 1-3-1-0 |
| MFLNH II | Monofucosyl-lacto-N-hexaose II | Galβ1-4(Fucα1-3)GlcNAcβ1-6(Galβ1-3GlcNAcβ1-3)Galβ1-4Glc | 1-4-2-0 |
| LNFP V | Lacto-N-fucopentaose V | Galβ1-3GlcNAcβ1-3Galβ1-4(Fucα1-3)Glc | 1-3-1-0 |
| LNnT | Lacto-N-neotetraose | Galβ1-4GlcNAcβ1-3Galβ1-4Glc | 0-3-1-0 |

| LNnH | Lacto-N-neohexaose | Galβ1-4GlcNAcβ1-6(Galβ1-4GlcNAcβ1-3)Galβ1-4Glc | 0-4-2-0 |
| --- | --- | --- | --- |
| LNT | Lacto-N-tetraose | Galβ1-3GlcNAcβ1-3Galβ1-4Glc | 0-3-1-0 |
| LNH | Lacto-N-hexaose | Galβ1-4GlcNAcβ1-6(Galβ1-3GlcNAcβ1-3)Galβ1-4Glc | 0-4-2-0 |
| FSLNnH | Fucosyl-sialyl-lacto-N-neohexaose | Galβ1-4(Fucα1-3)GlcNAcβ1-6(NeuAcα2-6Galβ1-4GlcNAcβ1-3)Galβ1-4Glc | 1-4-2-1 |
| LSTc | Lacto-sialyl tetrasaccharide c | NeuAcα2-6Galβ1-4GlcNAcβ1-3Galβ1-4Glc | 0-3-1-1 |
| 3'-SL | 3'-Sialyl-lactose | NeuAcα2-3Galβ1-4Glc | 0-2-0-1 |
| 6'-SL | 6'-Sialyl-lactose | NeuAcα2-6Galβ1-4Glc | 0-2-0-1 |
| LSTa | Lacto-sialyl tetrasaccharide a | NeuAcα2-3Galβ1-3GlcNAcβ1-3Galβ1-4Glc | 0-3-1-1 |
| LSTb | Lacto-sialyl tetrasaccharide b | NeuAcα2-6(Galβ1-3)GlcNAcβ1-3Galβ1-4Glc | 0-3-1-1 |
| LSTc | Lacto-sialyl tetrasaccharide c | NeuAcα2-6Galβ1-3GlcNAcβ1-3Galβ1-4Glc | 0-3-1-1 |
| DSLNT | Disialyl-lacto-N-tetraose | NeuAcα2-3Galβ1-3(NeuAcα2-6)GlcNAcβ1-3Galβ1-4Glc | 0-3-1-2 |
| F-LSTa | Fuco-lacto-sialyl tetrasaccharide a | NeuAcα2-3Galβ1-3(Fucα1-4)GlcNAcβ1-3Galβ1-4Glc | 1-3-1-1 |
| F-LSTb | Fuco-lacto-sialyl tetrasaccharide b | Fucα1-2Galβ1-3(NeuAcα2-6) GlcNAcβ1-3Galβ1-4Glc | 1-3-1-1 |
| F-LSTc | Fuco-lacto-sialyl tetrasaccharide c | NeuAcα2-6Galβ1-3GlcNAcβ1-3Galβ1-4(Fucα1-3)Glc | 1-3-1-1 |


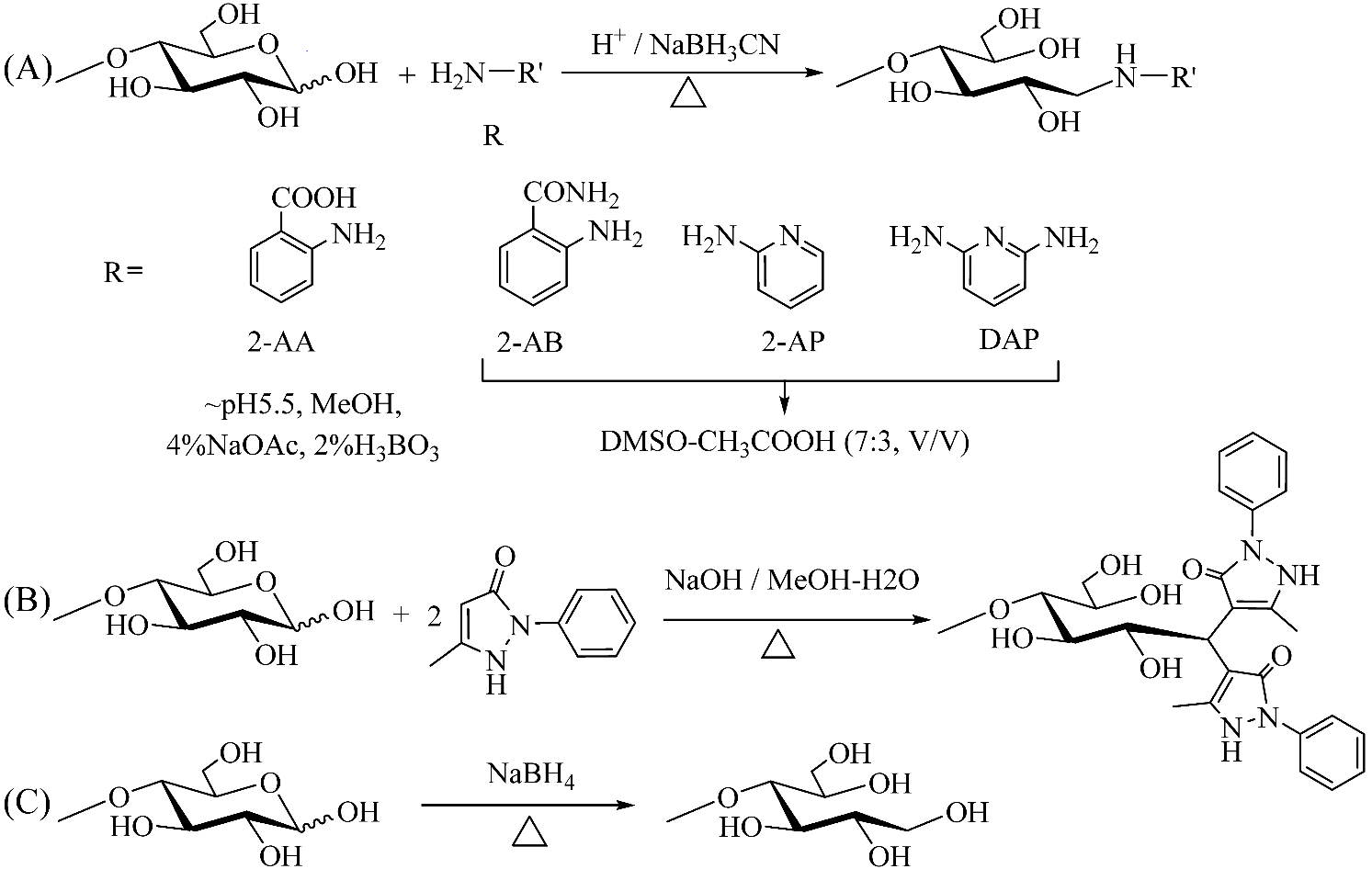


**Figure S1.** Derivatizations of HMOs. (**A**) 2-AA, 2-AB, 2-AP and DAP labeling via reductive amination; (**B**) PMP labeling via a Michael-type addition; and (**C**) reduction of HMOs into their alditols.


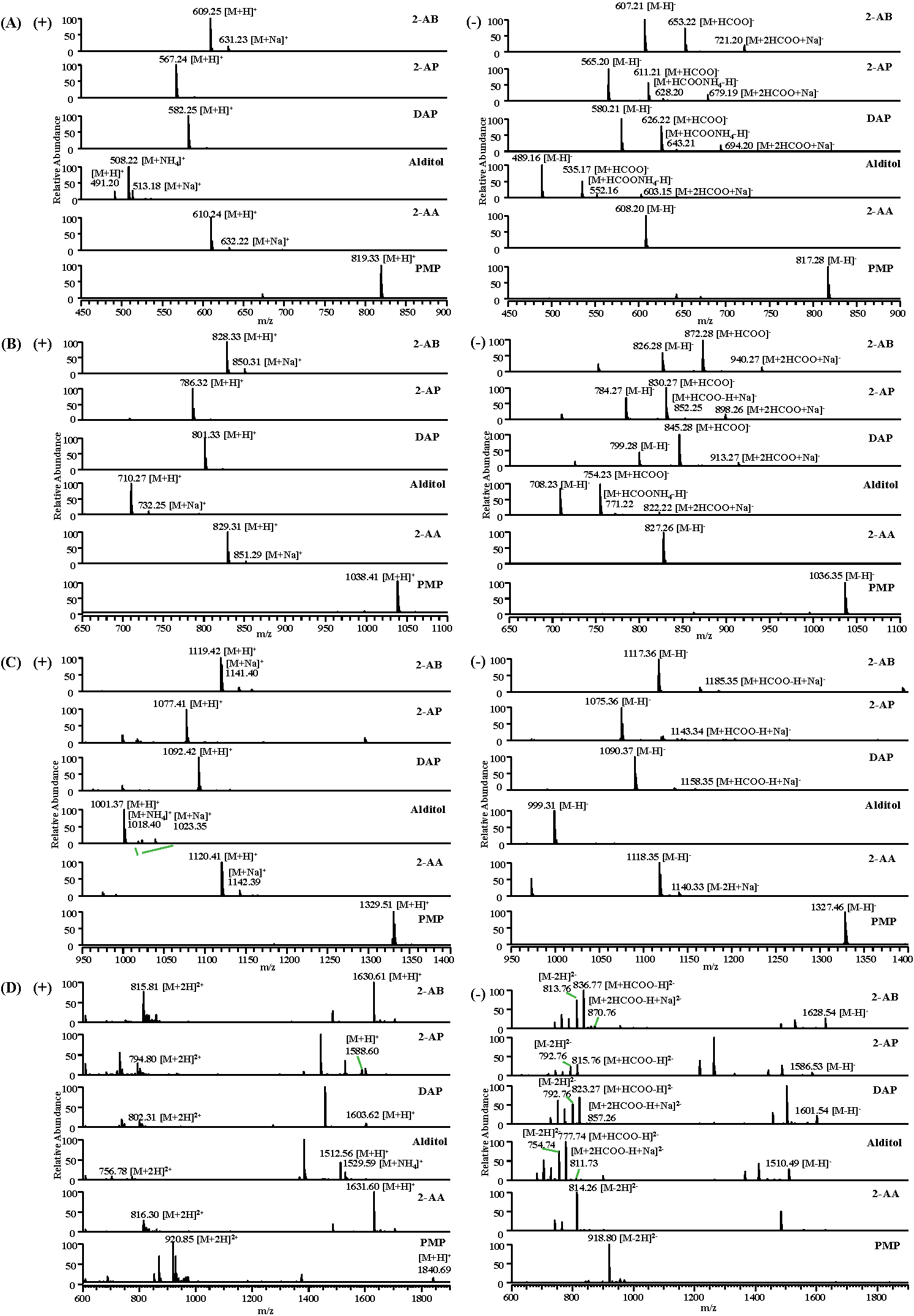


**Figure S2.** Positive-ion (**+**) and negative-ion (**-**) ESI-MS spectra of FL (**A**), LNT (**B**), LST (**C**) and MFMSLNH (**D**) derivatives of 2-AB, 2-AP, DAP, alditol, 2-AA and PMP.

**Table S2.** The major molecular ions for FL, LNT, LST and MFMSLNH derivatives of 2-AB, 2-AP, DAP, alditol, 2-AA and PMP produced from positive and negative ESI-MS.

| **Typical glycan** | **Positive mode** | | | | | | **Negative mode** | | | | | |
| --- | --- | --- | --- | --- | --- | --- | --- | --- | --- | --- | --- | --- |
|  | **2-AB** | **2-AP** | **DAP** | **Alditol** | **2-AA** | **PMP** | **2-AB** | **2-AP** | **DAP** | **Alditol** | **2-AA** | **PMP** |
| **FL** | [M+H]^+^ | | | [M+NH_4_]^2+^ | [M+H]^+^ | | [M–H]^–^/ [M+HCOO]^–^ (1.0-2.0) | | | | [M–H]^–^ | |
| **LNT** |  |  |  | [M+H]^+^ |  |  | [M–H]^–^/ [M+HCOO]^–^ (0.3-0.8) | | | |  |  |
| **LST** |  |  |  |  |  |  | [M–H]^–^ | | | |  |  |
| **MFMSLNH** | [M+2H]^2+^ | | | [M+H]^+^, [M+NH_4_]^2+^ | [M+2H]^2+^, [M+H]^+^ | | [M–H]^–^, [M–2H]^2–^,  [M + HCOO–H]^2–^ | | | | [M–2H]^2–^ | |


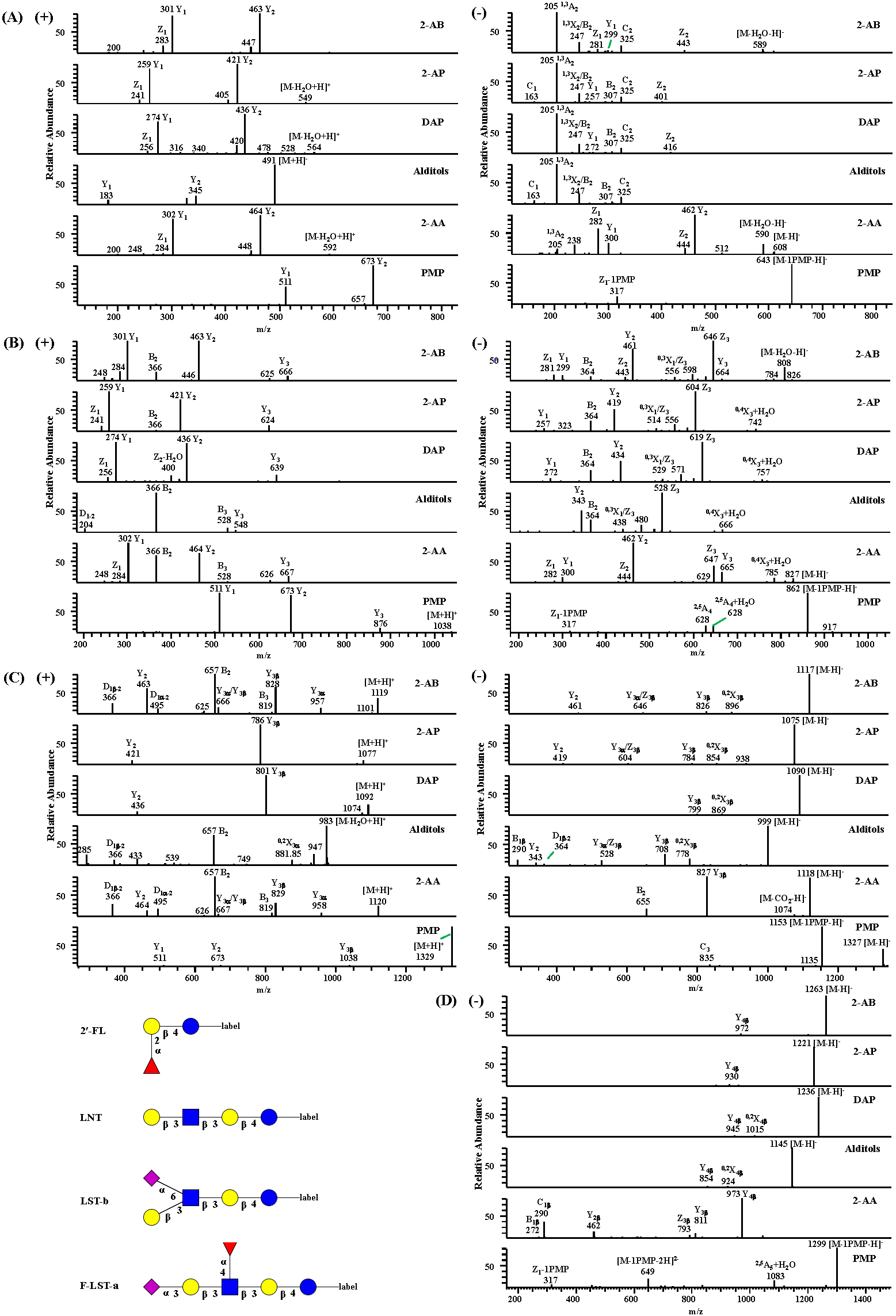


**Figure S3.** Positive-ion (+) and negative-ion (-) ESI-CID MS/MS spectra of 2'-FL (**A**), LNT (**B**), LST-b (**C**) and F-LST-a (**D**) detected in the reduced alditol form and derivatized with 2-AB, 2-AP, DAP, 2-AA and PMP.

**Table S3.** The predominant fragment patterns for 2'-FL, LNT, LST-b and F-LST-a derivatives of 2-AB, 2-AP, DAP, alditol, 2-AA and PMP produced from positive and negative ESI-CID-MS/MS.

| **Typical**  **glycan** | **Positive mode** | | | | | | **Negative mode** | | | | | |
| --- | --- | --- | --- | --- | --- | --- | --- | --- | --- | --- | --- | --- |
|  | 2-AB | 2-AP | DAP | Alditol | 2-AA | PMP | 2-AB | 2-AP | DAP | Alditol | 2-AA | PMP |
| **2**'**-FL** | Y | | | | | | ^1,3^A_2_, ^1,3^X_2_/B_2_, C_2_ | | | | Z, Y, ^1,3^A_2_, | [M-H-1PMP]^-^, Z_1_-1PMP |
| **LNT** | Y | | | B, D_1-2_ | B_2_, Y | Y | Y_2_, Z_3_, B_2_ (2-AB<2-AP<DAP) | | | | Z, Y |  |
| **LST-b** | Y, B, D, Y/Y | Y_3β_, Y_2_ | | B_2_, D_1β-2_, X_3α_ | Y, B, D, Y/Y | Y (minor) | Y_3β_ (minor), ^0,2^X_3β_ (minor) | | | Y, B, D, Y/Z, ^0,2^X_3β_ | Y_3β_, B_2_ (apparent) [M-H-CO_2_]^-^ |  |
| **F-LST-a** | Not detected | | | | | | Y_4β_ (minor), ^0,2^X_4β_ (minor) | | | | Z, Y, B, C (apparent) |  |


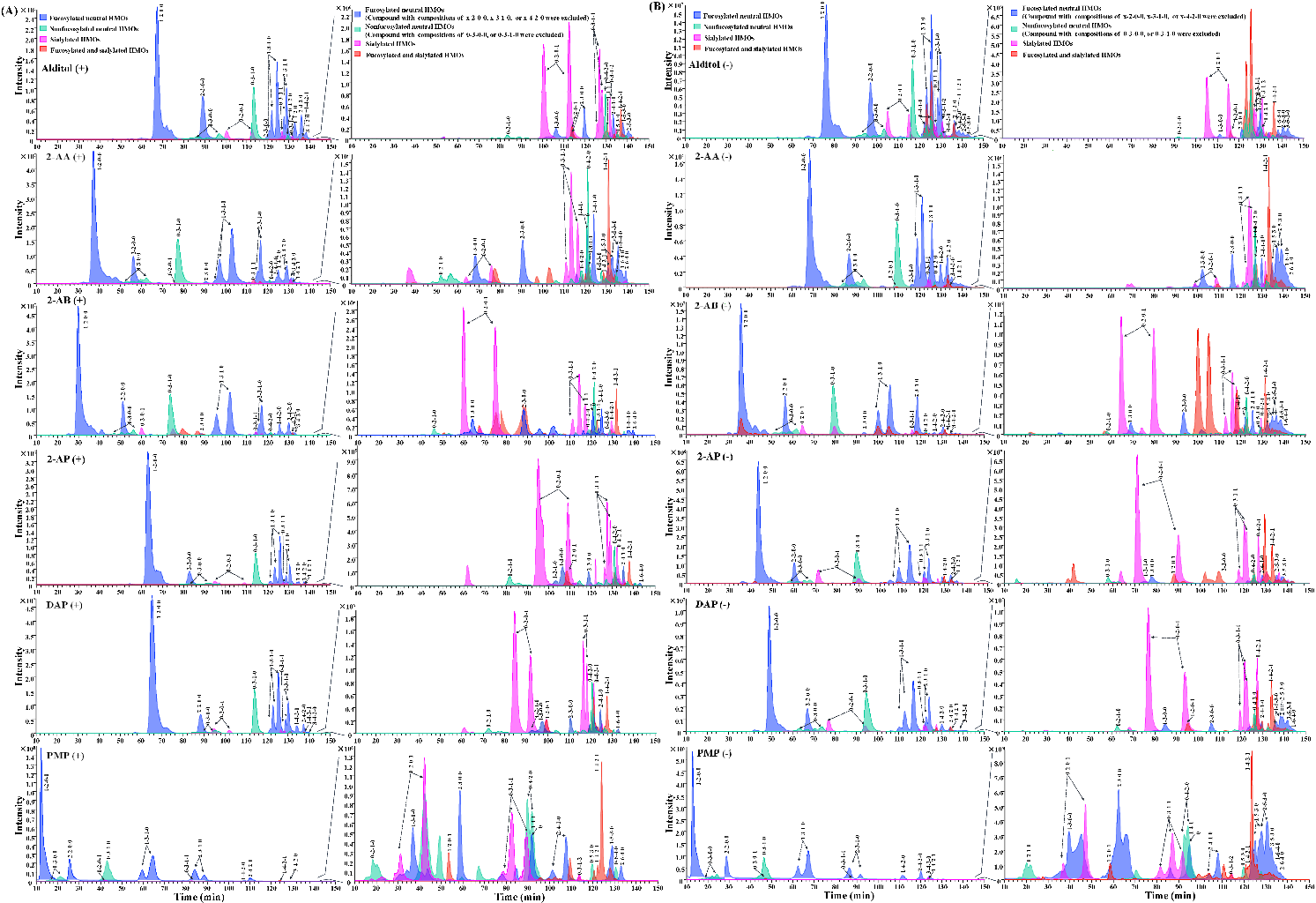


**Figure S4.** Overlaid extracted ion chromatograms (EIC) showing the elution profile of derivatized HMOs via HILIC-MS in positive (**A**) and negative (**B**) mode. Each glycan class is designated with a different color: fucosylated neutral (blue), nonfucosylated neutral (green), sialylated acidic (pink), and fucosylated and sialylated acidic (red). Figures on the right side of the panels give the profiles of the peak of low abundances (compositions of x-2-0-0, x-3-1-0, x-4-2-0, 0-3-0-0 and 0-3-1-0 were excluded).

**Table S4.** The counts of visible EIC peaks for each monosaccharide composition obtained from three analytical protocols: protocol 1, protocol 2 and protocol 3.

| **Glycan compound**  (Fuc-Hex-HexNac-NeuAc) | **Visible EIC peaks acquired from three protocols** | | |
| --- | --- | --- | --- |
|  | **Protocol 1** | **Protocol 2** | **Protocol 3** |
| 1-2-0-0 | 1 | 3 | 3 |
| 2-2-0-0 | 1 | 1 | 1 |
| 1-3-1-0 | 2 | 4 | 4 |
| 2-3-1-0 | 1 | 3 | 3 |
| 3-3-1-0 | 1 | 1 | 1 |
| 1-4-2-0 |  | 4 | 2 |
| 2-4-2-0 |  | 4 | 4 |
| 3-4-2-0 | 3 | 5 | 5 |
| 1-5-3-0 | 1 | 3 | 3 |
| 2-5-3-0 | 1 | 9 | 7 |
| 3-5-3-0 | 1 | 4 | 4 |
| 1-6-4-0 | 1 |  | 2 |
| 2-6-4-0 | 1 | 3 | 2 |
| 0-3-1-0 | 1 | 2 | 2 |
| 0-4-2-0 | 1 | 3 | 3 |
| 0-5-3-0 | 1 | 4 | 2 |
| 0-6-4-0 |  | 2 | 2 |
| 0-2-0-1 | 2 | 2 | 2 |
| 0-3-1-1 | 3 | 3 | 3 |
| 0-3-1-2 | 1 |  | 4 |
| 0-4-2-1 | 2 | 1 | 1 |
| 0-4-2-2 |  |  | 1 |
| 1-2-0-1 |  |  | 2 |
| 1-3-1-1 | 2 | 4 | 3 |
| 1-4-2-1 | 1 | 6 | 4 |
| 2-4-2-1 | 2 | 2 | 1 |
| 1-4-2-2 |  |  | 1 |
| 1-5-3-1 | 1 | 1 | 1 |
| 2-5-3-1 | 1 |  | 1 |
| Total peaks that can be quantifiable | 32 | 74 | 74 |
| Total monosaccharide compositions that can be quantifiable | 23 | 23 | 29 |


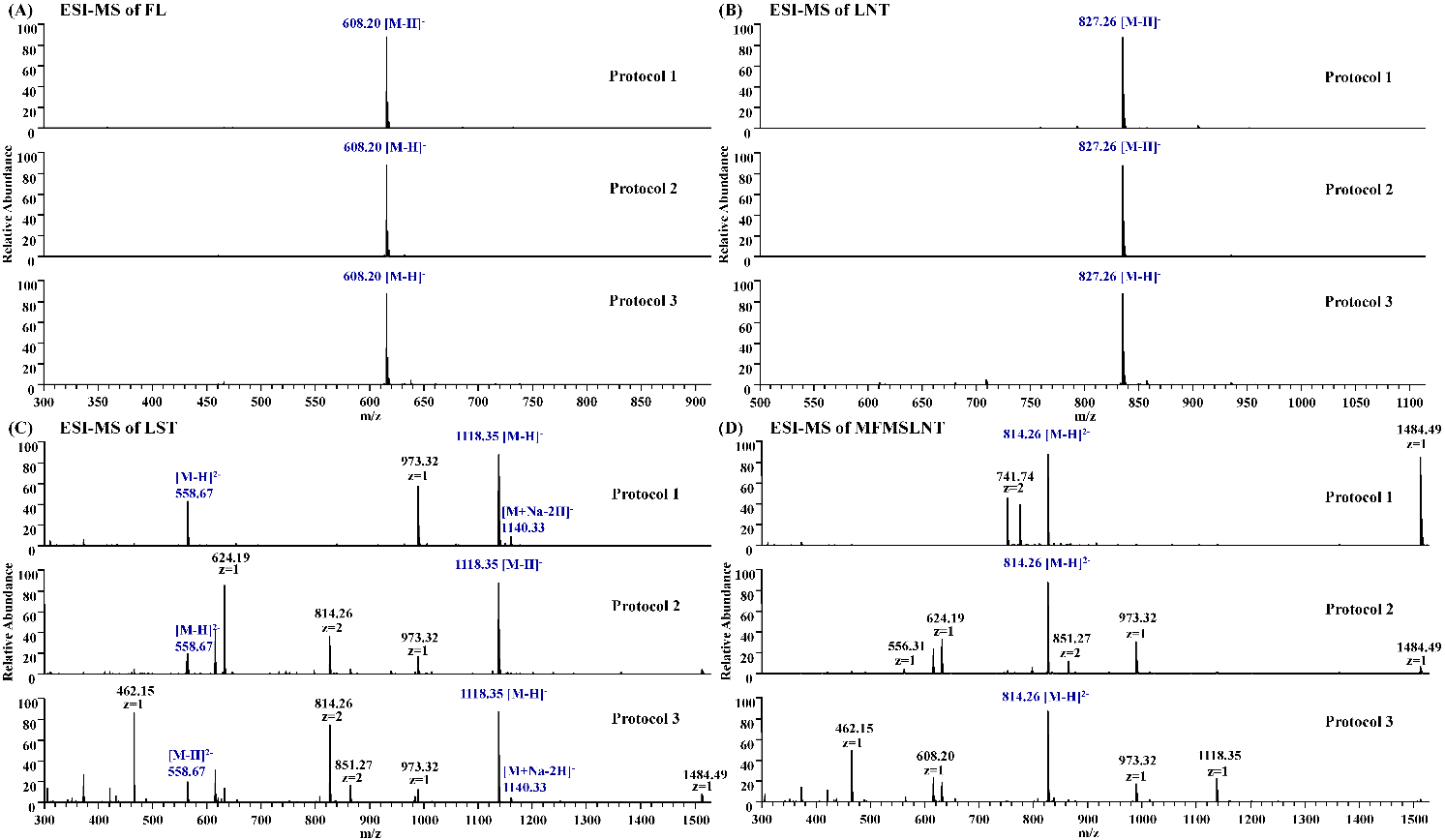


**Figure S5.** Negative-ion (**-**) ESI-MS spectra of FL (**A**), LNT (**B**), LST (**C**) and MFMSLNH (**d**) derivatives of 2-AA in version 1, version 2 and version 3.


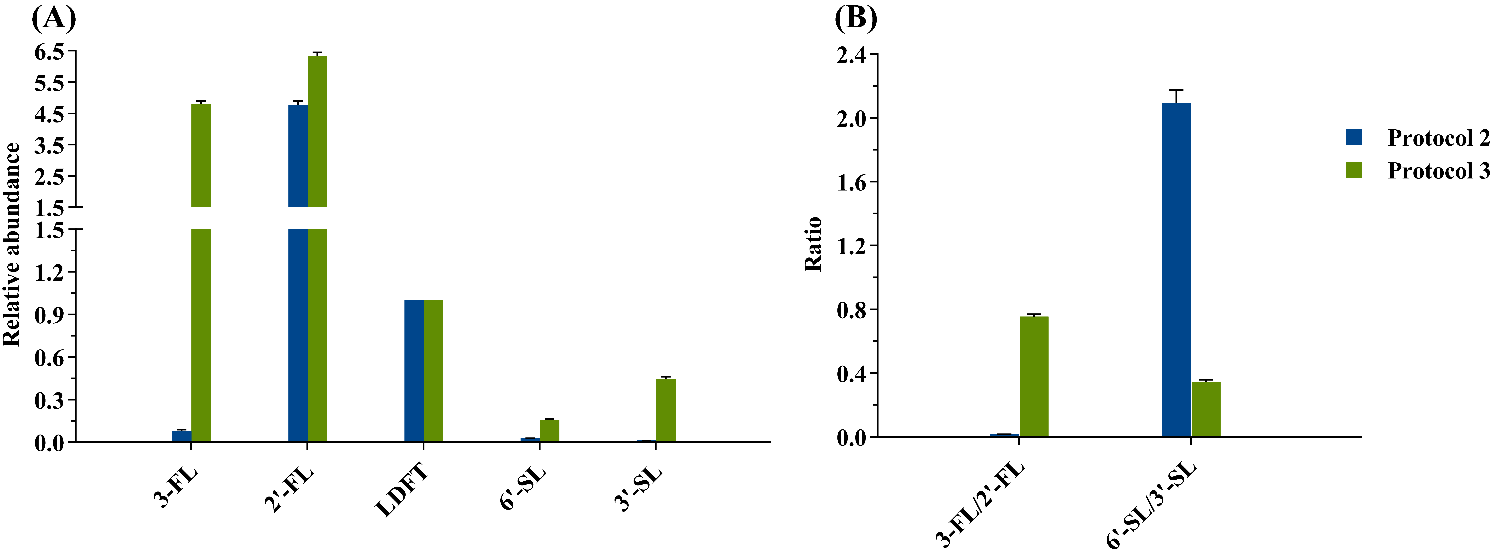


**Figure S6.** (A) Comparison of the relative abundance of FL isomers and SL isomers in version 2 and version 3 (LDFT was selected as the internal standard); (B) Comparison of isomeric ratio of 3-FL/2ꞌ-FL and 6ꞌ-SL/3ꞌ-SL in version 2 and version 3.
